# The timing of auditory sensory deficits in Norrie disease has implications for therapeutic intervention

**DOI:** 10.1172/jci.insight.148586

**Published:** 2022-02-08

**Authors:** Dale Bryant, Valda Pauzuolyte, Neil J. Ingham, Aara Patel, Waheeda Pagarkar, Lucy A. Anderson, Katie E. Smith, Dale A. Moulding, Yeh C. Leong, Daniyal J. Jafree, David A. Long, Amina Al-Yassin, Karen P. Steel, Daniel J. Jagger, Andrew Forge, Wolfgang Berger, Jane C. Sowden, Maria Bitner-Glindzicz

**Affiliations:** 1 UCL Great Ormond Street Institute of Child Health, University College London, and NIHR Great Ormond Street Hospital Biomedical Research Centre, London, United Kingdom.; 2 Wolfson Centre for Age-Related Diseases, King’s College London, London, United Kingdom.; 3 Great Ormond Street Hospital, Great Ormond Street, London, United Kingdom.; 4 UCL Ear Institute, University College London, London, United Kingdom.; 5 UCL MB/PhD Programme, Faculty of Medical Sciences, University College London, London, United Kingdom.; 6 Institute of Medical Molecular Genetics, University of Zürich, Schlieren, Switzerland.; 7 Neuroscience Center Zurich, University and ETH Zurich, Zurich, Switzerland.; 8 Zurich Center for Integrative Human Physiology (ZIHP), University of Zurich, Zurich, Switzerland.

**Keywords:** Development, Otology, Endothelial cells, Genetic diseases, Mouse models

## Abstract

Norrie disease is caused by mutation of the *NDP* gene, presenting as congenital blindness followed by later onset of hearing loss. Protecting patients from hearing loss is critical for maintaining their quality of life. This study aimed to understand the onset of pathology in cochlear structure and function. By investigating patients and juvenile *Ndp*-mutant mice, we elucidated the sequence of onset of physiological changes (in auditory brainstem responses, distortion product otoacoustic emissions, endocochlear potential, blood-labyrinth barrier integrity) and determined the cellular, histological, and ultrastructural events leading to hearing loss. We found that cochlear vascular pathology occurs earlier than previously reported and precedes sensorineural hearing loss. The work defines a disease mechanism whereby early malformation of the cochlear microvasculature precedes loss of vessel integrity and decline of endocochlear potential, leading to hearing loss and hair cell death while sparing spiral ganglion cells. This provides essential information on events defining the optimal therapeutic window and indicates that early intervention is needed. In an era of advancing gene therapy and small-molecule technologies, this study establishes *Ndp*-mutant mice as a platform to test such interventions and has important implications for understanding the progression of hearing loss in Norrie disease.

## Graphical abstract

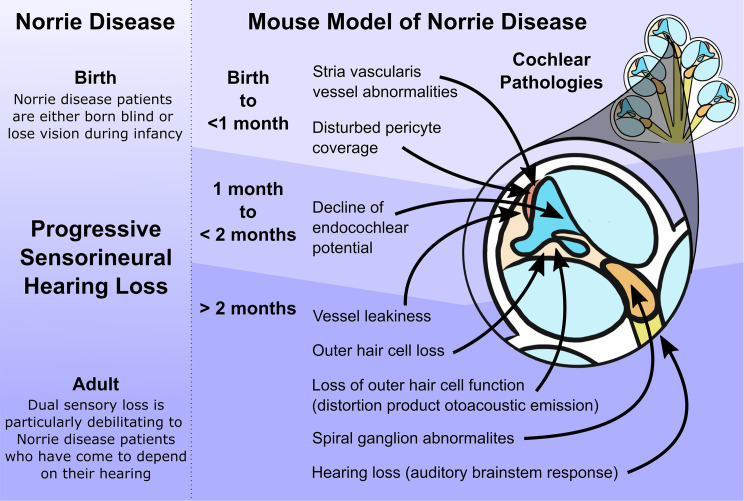

## Introduction

Norrie disease is an X-linked disorder caused by mutations in the Norrie disease pseudoglioma (*NDP*) gene (1). It results in congenital or neonatal bilateral blindness resulting from pathological development and detachment of the retina (2). Norrie disease is also characterized by extraocular phenotypes including sensorineural hearing loss, cognitive impairment, behavioral disturbances, seizures, and peripheral vascular disease (3–5). There appears to be normal hearing ability from birth with deterioration reported from as early as 5 and as late as 48 years of age (5). Deterioration is particularly debilitating as patients with Norrie disease grow increasingly dependent on their remaining hearing in the absence of vision.

In contrast to the congenital nature of the blindness, the childhood/adult onset of hearing loss does provide a possible opportunity to intervene and prevent it from happening. To achieve this, we must first understand the sequence of events underlying the progression of hearing loss in Norrie disease. Given that Norrie disease is rare and it is difficult to examine cochlear pathology prior to death, investigations into the events leading to hearing loss in patients have been limited. In 1990, Nadol et al. described observations from postmortem tissue of a 77-year-old Norrie disease patient who suffered from hearing loss. They reported effects in the whole cochlea with a severely atrophied stria vascularis (the ion-transporting vascularized epithelium lining the cochlear lateral wall), loss of sensory hair cells throughout the cochlea, and dense deposits of fibrous tissue around degenerating spiral ganglion neurons (SGNs; they normally synapse with hair cells and project axons along the auditory nerve) (6). Clinical studies have reported that auditory function is first affected in the high frequencies, with often only mild, asymmetric hearing loss (4, 5, 7, 8) and auditory brainstem response (ABR) thresholds that are skewed or boat-shaped, meaning that particular frequency-specific sites are affected more than others (4, 5, 7, 9). One study of 3 patients with Norrie disease in their 30s showed normal hair cell and auditory brainstem function, but a cochlear lesion at the SGN–auditory nerve level, as implicated from the abnormal whole-nerve action potential (7). Conversely, some authors predict that patient-reported “stuffiness” at the onset of hearing loss, tinnitus, and temporary, fluctuating, intermittent hearing loss (5) could signify temporary abnormalities in the endolymphatic fluid composition at the onset of the disease, leading to endocochlear potential (EP) abnormalities or endolymphatic hydrops (ballooning of the endolymph fluid) (5, 8, 10).

Mice with a loss-of-function mutation in the *Ndp* gene (*Ndp^tm1Wbrg/Y^*) display an ocular phenotype consistent with that of patients with Norrie disease, albeit less severe (11, 12). A previous study of the cochlea of *Ndp^tm1Wbrg/Y^* mice examined histology and hearing loss (by ABRs to pure tone bursts) in aging mice from 3 to 15 months but did not analyze auditory function at earlier stages. The *Ndp^tm1Wbrg/Y^* mice showed progressive hearing loss and pathological changes in the cochlea including enlarged blood vessels in the stria vascularis, loss of hair cells, and degeneration of the spiral ganglion (10). The anomalies in stria vascularis are reminiscent of the abnormal blood vessel development in the retina (6, 10, 11, 13). This suggests a common mechanism driving the pathology of Norrie disease in the cochlea and in the eye. However, it is important that the onset of these pathologies is defined so that it can guide therapeutic interventions.

Exactly how mutations in *NDP* result in hearing loss remains uncertain. NDP is a secreted protein that has affinity for the frizzled-4 (FZ4) receptor, which, together with the coreceptor LDL receptor–related protein 5 (LRP5) and the membrane protein tetraspanin 12 (TSPAN12), regulates vasculature development in the retina via canonical WNT signaling (13–16). Indeed, mutations in genes that encode FZ4 (17), LRP5 (18), and TSPAN12 (19) cause familial exudative vitreoretinopathy (FEVR), in which blood vessel development is disrupted in the retina. Given that *Fz4*-mutant mice also display dilated vessels in the stria vascularis (14), it seems likely that WNT signaling via the NDP/FZ4/LRP5/TSPAN12 pathway also accounts for the stria vascularis vessel abnormalities in Norrie disease.

Before effective preclinical interventions aimed at preventing hearing loss in Norrie disease can be designed, the nature and the timing of the onset of cochlear pathology need to be determined. Therefore, the goal of this study was to carefully define the onset of the hearing loss, and to explain its relationship with the described cellular pathologies. We describe the presentation of hearing loss in children with Norrie disease and an analysis of early changes in cochlear function and structure in the genetic mouse model of Norrie disease. We report abnormalities in development of the cochlear microvasculature by postnatal day 10 (P10), much earlier than the onset of hearing abnormalities, in *Ndp*-mutant mice. These results are important for the design of preclinical investigations, as they provide description of the initial onset of events leading to hearing loss.

## Results

### Onset of hearing loss in Norrie disease.

We studied available audiograms from 6 patients with Norrie disease and hearing loss. Onset of hearing loss was detected at the ages of 3 years (N01), 6 years (N05), and 8 years (N07). For the patients whose hearing loss was detected at the ages of 4 years (N08), 5 years (N04), and 35 years (N06), we had no earlier audiograms, although N04 passed the newborn hearing screen. Hence the onset of hearing loss could have been earlier.

**Figure 1:**
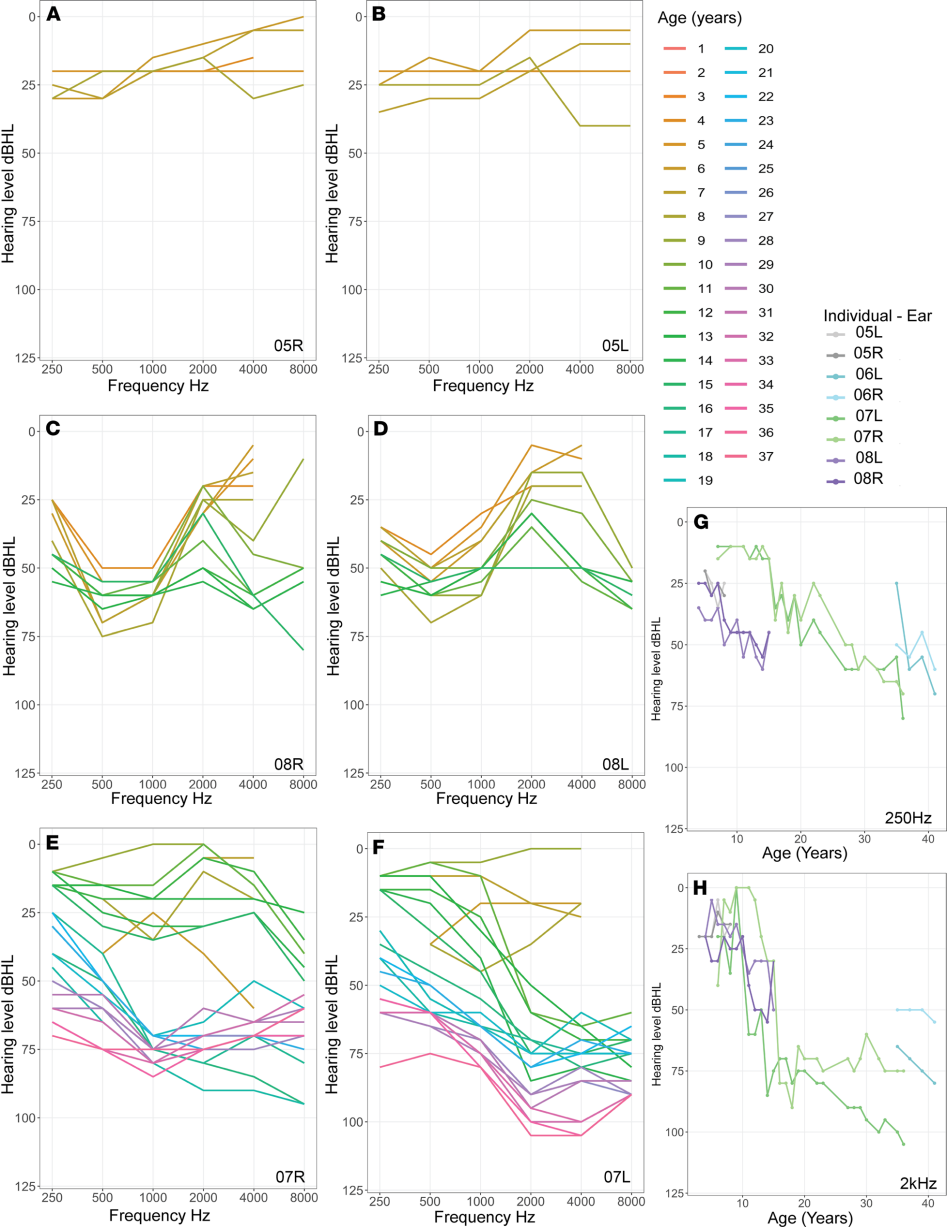
Sequential audiograms of patients with Norrie disease showing onset of hearing loss. (**A**–**F**) Hearing levels at a range of frequencies and multiple ages for each individual. (**A** and **B**) Audiograms of right and left ears of the patient (N05) between 3 and 8 years showing onset of mild hearing loss at low frequencies at age 6. Note a progression each year in high frequencies. (**C** and **D**) Audiograms of right and left ears of the patient (N08) between 4 and 19 years. Note the presence of hearing loss at 4 years of age, and the tented shape and gradual progression across frequencies over the years. (**E** and **F**) Audiograms of right and left ears of the patient (N07) between 6 and 36 years showing onset of hearing loss at age 8 years, normal levels at 9 years, and subsequent drop in hearing at 11 years, affecting the right ear first. Note the skew toward loss of high frequencies and the marked jump of thresholds in adolescence in this patient. (**G** and **H**) Change over time in hearing levels at selected, speech-relevant, frequencies in 4 individuals.

Figure 1 shows onset and progressive hearing loss with some fluctuation over time for 3 children (N05, N07, N08) for whom multiple sequential audiograms were available. In one patient (N05) (Figure 1, A and B), hearing was within normal limits at age 4, with a mild low-frequency loss initially on the left, and in both ears by age 6. By age 8, high-frequency hearing was also affected bilaterally. In this child, otoacoustic emissions (OAEs) had been reliably recorded indicating that the outer hair cell (OHC) function in both ears was not affected, despite the child having a mild hearing loss.

In another patient (N08), for whom audiograms were available for 14 years (ages 4–18 years), hearing loss was present at the age of 4 years (Figure 1, C and D). The low and mid-frequencies were affected at onset. The high frequencies were shown to be affected from the age of 8 years onward, although the highest frequency of 8 kHz was not tested before this age, and could have been earlier affected. A flattish/tented configuration was evident from the age of 12 years onward.

The longest duration of follow-up of an individual patient (N07) was 30 years (6–36 years of age) (Figure 1, E and F). Normal hearing was preserved until 8 years of age, when the hearing loss was detected. This child subsequently had normal hearing documented at 9 years of age, following which a hearing loss was again documented at 11 years of age. This could signify a fluctuation of hearing before progressive hearing loss is evident. There was no middle ear dysfunction to account for this fluctuation. The hearing loss affected high frequencies in the right ear first (Figure 1E), initially giving the appearance of a unilateral hearing loss. The left ear was affected with progressive loss of sensitivity (Figure 1F). The high-frequency configuration of the hearing loss was preserved until 20 years of age, after which the configuration flattened. By the age of 36 years this patient had a severe to profound loss in the right ear and a moderate to severe loss in the left (Figure 1, E and F).

The hearing loss was asymmetric at onset in 3 of 6 patients with a trend toward the same flattish configuration over time. The degree of hearing loss as a function of time followed a similar trajectory between patients, both at low frequencies (250 Hz; Figure 1G) and at mid-frequencies (2 kHz; Figure 1H) within the speech range. In 2 patients whose latest audiograms were at ages 36 and 41 years (N07, N06), the hearing loss was moderate to profound. However, a similar degree of hearing loss was also detected in one 5-year-old (N05), with profound hearing loss in the left ear. Four of the six patients wore hearing aids. None had cochlear implants.

These clinical data showed that hearing loss may affect high or low frequencies first and it then progresses to other frequencies (Figure 1). This type of data is not sufficient to confirm the exact site of the pathology (i.e., cochlear, retrocochlear, exact site in cochlea affected) or the extent of pathology affecting the cochlea and spiral ganglion cells (first-order auditory neurons).

### Early onset of auditory abnormalities in Ndp-KO mice.

**Figure 2:**
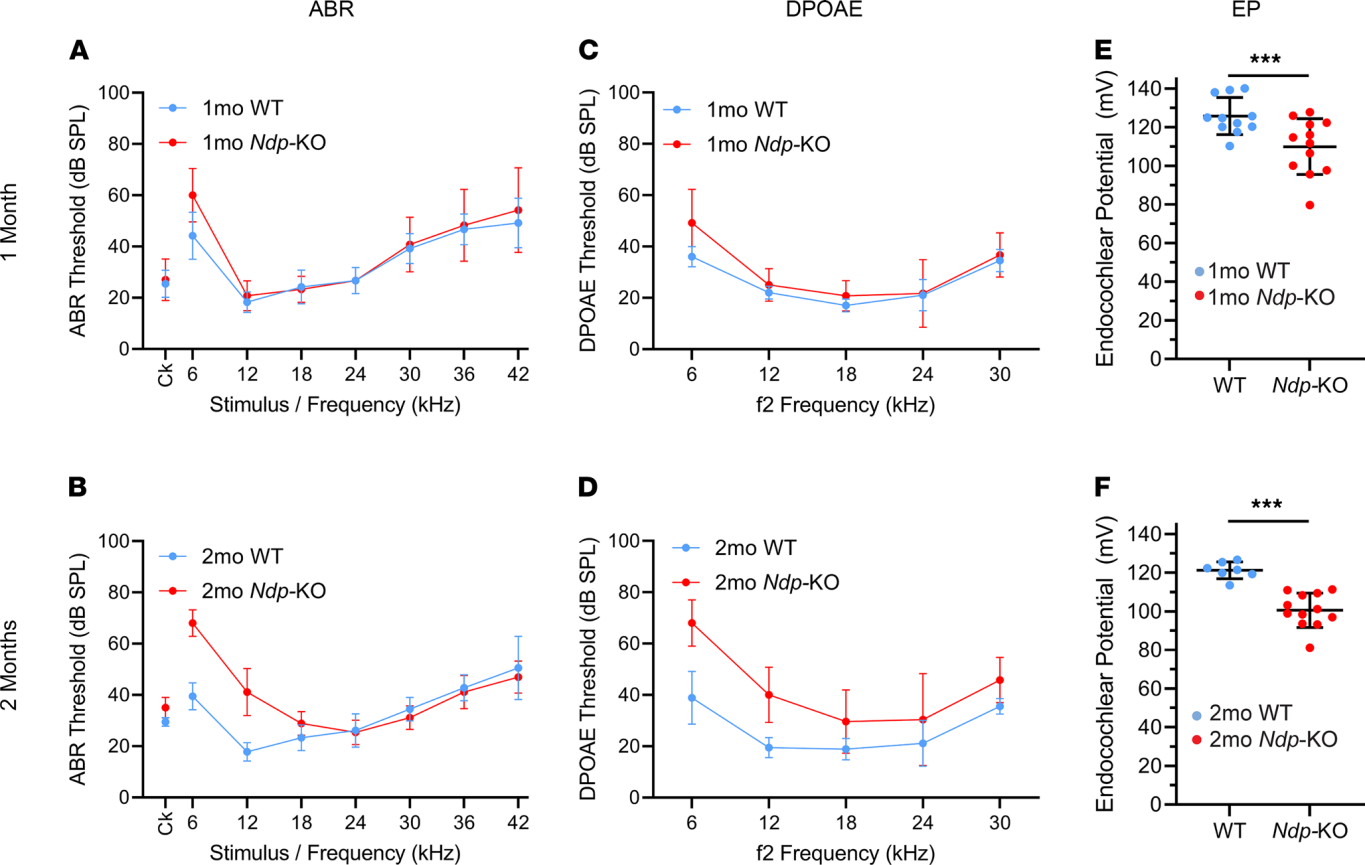
Early onset of hearing impairment in *Ndp-*KO mice. ABR (**A** and **B**) and DPOAE (**C** and **D**) thresholds and endocochlear potentials (EPs) (**E** and **F**) for individual animals were plotted as mean ± SD from WT mice (blue) and *Ndp-*KO mice (red) aged 1 month and 2 months. Ck, click. (**A**) No difference in ABR thresholds between genotypes at 1 month (Mann-Whitney rank sum test; *U* = 1050, *T* = 2226, *P* = 0.4540). (**B**) *Ndp-*KO mice show hearing loss in low frequencies at 2 months (*U* = 2754.4, *T* = 5382.5, *P* = 0.0026). *n* = 11 WT, *n* = 12 *Ndp*-KO at 1 month; *n* = 9 WT, *n* = 13 *Ndp*-KO at 2 months. (**C** and **D**) DPOAEs were comparable between genotypes at 1 month (*U* = 1252.5, *T* = 2527.5, *P* = 0.1326). At 2 months, *Ndp-*KO thresholds increased over all frequencies compared with controls (*U* = 689.5, *T* = 1724.5, *P* < 0.00000001). *n* = 10 WT, *n* = 12 *Ndp*-KO at 1 month; *n* = 9 WT, *n* = 13 *Ndp*-KO at 2 months. (**E**) At 1 month, EP was significantly lower in *Ndp-*KO than in WT, but within normal range (>100 mV); *n* = 11 WT, *n* = 12 *Ndp-*KO. (**F**) At 2 months, *Ndp-*KO EP decreased further; *n* = 7 WT, *n* = 12 *Ndp-*KO (2-way ANOVA; significant effect of age, *F* = 4.3400, *P* = 0.0440; significant effect of genotype, *F* = 29.9970, *P* = 0.0000297). There was a significant reduction at 1 month (**E**) (Holm-Šidák method for multiple comparisons; *t* = 3.5946, *P* = 0.00092067) and at 2 months (**F**) (Holm-Šidák method for multiple comparisons; *t* = 4.1323, *P* = 0.00019031). *n* = 11 WT, *n* = 12 *Ndp-*KO at 1 month; *n* = 7 WT, *n* = 12 *Ndp*-KO at 2 months.

To better understand the presentation of hearing loss in Norrie disease, we conducted a detailed analysis of early changes in cochlear function in mice with the Norrie disease gene inactivated (*Ndp*-KO). ABRs provide an objective measure of auditory function and were used here to assess whether the Norrie disease mouse mutant showed abnormalities in auditory function at 1 month (juveniles) and 2 months old (adults) (Figure 2). Anesthetized animals were presented with clicks or pure tone stimuli across a range of stimulus levels, and the averaged ABRs were detectable as a series of voltage waveforms from which a threshold sensitivity for each stimulus was estimated. At 1 month there were no significant increases in ABR thresholds (measured in decibels sound pressure level; dB SPL) in *Ndp-*KO mice (Mann-Whitney rank sum test; *U* = 1050, *T* = 2226, *P* = 0.4540) (Figure 2A). By 2 months, the pathology had progressed and ABR thresholds of *Ndp-*KO mice were significantly higher than those of WT mice (Mann-Whitney rank sum test; *U* = 2754.4, *T* = 5382.5, *P* = 0.0026), with elevated thresholds apparent for clicks and 6- to 18-kHz tones (apical), but not for higher-frequency stimuli (Figure 2B).

Further examination revealed that the click-evoked ABR wave I was significantly reduced in amplitude in *Ndp-*KO compared with WT at 1 month (Mann-Whitney rank sum test; *U* = 7996, *T* = 29,041, *P* < 0.00000001) (Supplemental Figure 1A; supplemental material available online with this article; https://doi.org/10.1172/jci.insight.148586DS1). However, this difference was not seen in the 2-month-old group (Mann-Whitney rank sum test; *U* = 9312.5, *T* = 16,895.5, *P* = 0.6148) (Supplemental Figure 1C). Conversely, ABR wave III was comparable in amplitude between the 2 genotypes at 1 month (Mann-Whitney rank sum test; *U* = 11,266, *T* = 23,201, *P* = 0.2588) (Supplemental Figure 1B) but was significantly reduced in the *Ndp*-KO by 2 months (Mann-Whitney rank sum test; *U* = 6501, *T* = 19,707, *P* < 0.00000001) (Supplemental Figure 1D). The latencies of ABR waves I and III were comparable between WT and *Ndp-*KO mice at both time points (Mann-Whitney rank sum test; *P* > 0.05 in each comparison) (Supplemental Figure 1, E–H). A similar ABR data set obtained on a separate cohort of 1-month-old mice, recorded in a different laboratory, confirmed the reduced wave I amplitude in *Ndp-*KO compared with WT at 1 month (data not shown).

These data demonstrate that hearing abnormalities were detectable in juvenile and young adult *Ndp-*KO mice compared with WT, and the wave I deficit was only found in the 1-month-old group. Adult-like auditory responses are normally achieved by the end of the first postnatal month (20).

### Reduced function of OHCs in Ndp-KO mice.

We next assessed OHC function at 1 and 2 months using distortion product otoacoustic emissions (DPOAEs) (Figure 2, C and D). At 1 month old the DPOAE thresholds of *Ndp*-KO mice were similar to the WT (Mann-Whitney rank sum test; *U* = 1252.5, *T* = 2527.5, *P* = 0.1326) (Figure 2C). By 2 months, DPOAE thresholds were significantly elevated (Mann-Whitney rank sum test; *U* = 689.5, *T* = 1724.5, *P* < 0.00000001), with thresholds showing elevations across all f2 frequencies tested from 6 to 30 kHz (Figure 2D) compared with WT, indicating abnormal OHC function in the *Ndp* mutants. Thresholds were calculated from 2f1–f2 DPOAE amplitudes plotted as a function of decibels sound pressure level. When these functions were corrected for threshold shift (dB SPL), the slope of DPOAE growth was comparable for each f2 stimulus in each age group (Supplemental Figure 2, A–D, illustrates this using f2 stimuli of 6 kHz and 18 kHz).

### Reduced endocochlear potential in Ndp-KO mice.

As endocochlear potential (EP; the high resting potential in the endolymph bathing the hair cell stereocilia on the surface of the organ of Corti) is essential for sound transduction, we assessed whether EP deviated from normal levels at 1 and 2 months. A rise in EP can be first measured by P6 (21) and then normally increases from approximately +20 mV until it reaches a stable value around +100 to +120 mV by P20. Measurements were performed in the basal turn of the cochlea, close to the round window. Significant differences were found between EPs measured in WT and *Ndp*-KO mice at both ages (2-way ANOVA; significant effect of age, *F* = 4.3400, *P* = 0.0440; significant effect of genotype, *F* = 29.9970, *P* = 0.0000297). At 1 month, EP was significantly reduced from 125.75 ± 9.62 mV in WT to 109.96 ± 14.47 mV in *Ndp*-KO mice, but at its mean value of 110 mV, there would be little effect on cochlear function expected (Holm-Šidák method for multiple comparisons; *t* = 3.5946, *P* = 0.00092067) (Figure 2E). At 2 months, EP was reduced further relative to WT (121.26 ± 4.35 mV) to an average of 100.57 ± 8.90 mV in the *Ndp*-KO mutants (Holm-Šidák method for multiple comparisons; *t* = 4.1323, *P* = 0.00019031) (Figure 2F), a level at which cochlear function might be expected to become impaired. Interestingly, there was a significant reduction in EP over time measured from *Ndp*-KO mice aged 1 month compared with those aged 2 months (Holm-Šidák method for multiple comparisons; *t* = 2.1829, *P* = 0.03521), whereas the EP of WT mice was not significantly changed (Holm-Šidák method for multiple comparisons; *t* = 0.8826, *P* = 0.3830). EP reduction in the *Ndp* mutants therefore preceded the abnormal OHC function (DPOAEs) and elevated ABR thresholds.

### Analysis of the SGNs in Ndp-KO mice.

**Figure 3:**
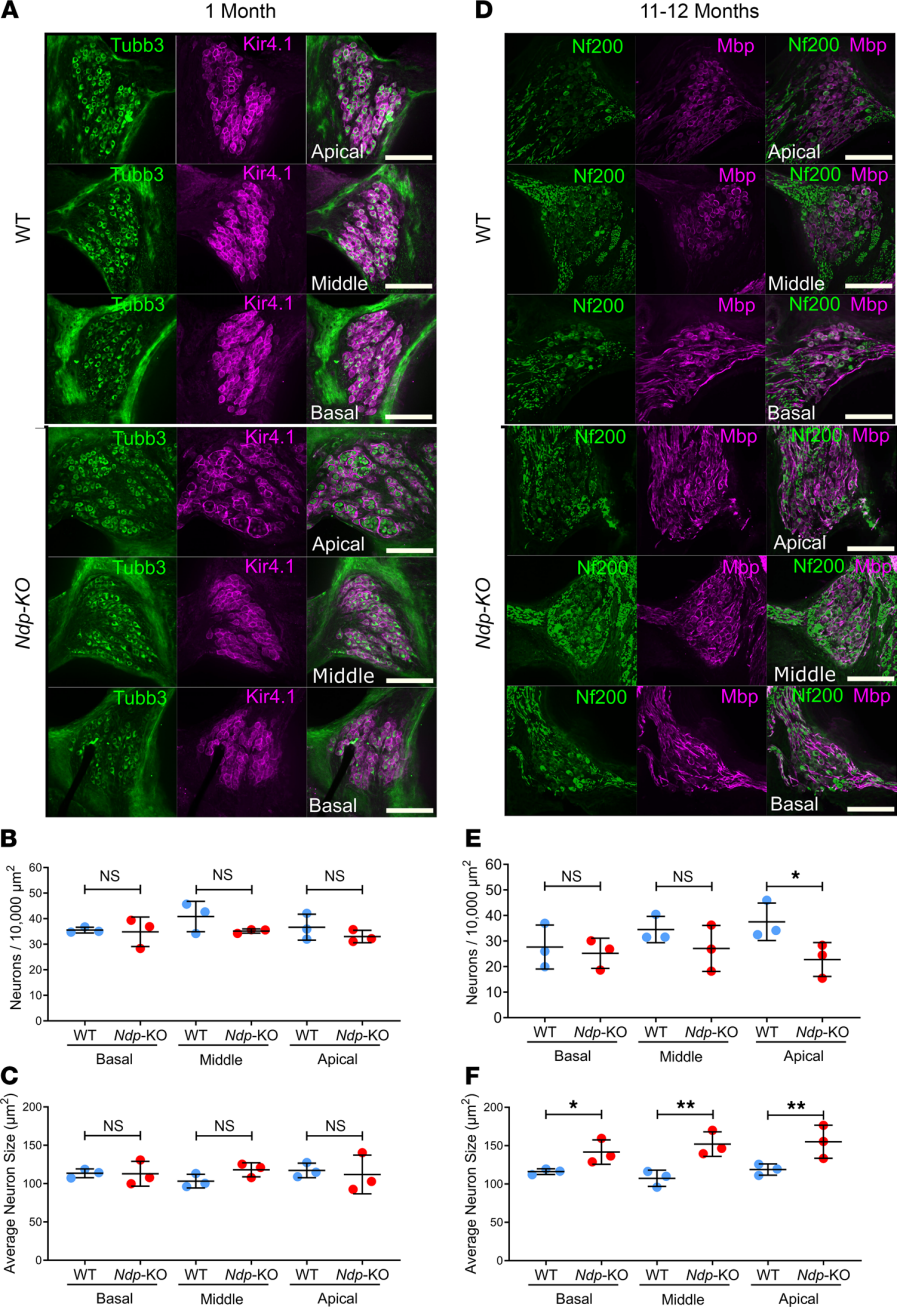
SGN survival in *Ndp-*KO mice. (**A**) Apical to basal spiral ganglia of WT and *Ndp*-KO mice at 1 month showing that Tubb3 is expressed in the SGNs and Kir4.1 is expressed in the surrounding satellite glial cells. (**B**) Number of Tubb3-positive SGNs per 10,000 μm^2^ in apical to basal spiral ganglia of WT and *Ndp*-KO mice at 1 month. (**C**) Average size (μm^2^) of SGNs in apical to basal spiral ganglia of WT and *Ndp*-KO mice at 1 month. (**D**) Apical to basal spiral ganglia of WT and *Ndp*-KO mice at 11–12 months showing that Nf200 is expressed in the spiral SGNs and Mbp is expressed in the surrounding satellite glial cells. (**E**) Number of Nf200-positive SGNs per 10,000 μm^2^ in apical to basal spiral ganglia of WT and *Ndp*-KO mice at 11–12 months. (**F**) Average size (μm^2^) of SGNs in apical to basal spiral ganglia of WT and *Ndp*-KO mice at 11–12 months. High-magnification images of SGNs are shown in Supplemental Figure 3. *n* = 3 WT, *n* = 3 *Ndp-*KO analyzed at each time point; bars indicate mean ± SD. Analyzed with 1-way ANOVA, Holm-Šidák correction for multiple comparisons; *P ≤ 0.05, **P ≤ 0.01; NS, *P* ≥ 0.05. Scale bars: 100 μm.

Given the decreased ABR wave I amplitudes in *Ndp-*KO mice at the end of the first postnatal month, we first examined the SGNs to assess whether there was a corresponding loss of these primary afferent neurons. A previous study reported loss of SGNs from the apical cochlear region in 15-month-old *Ndp-*KO mice (10). We used immunostaining to label the SGNs and the satellite glial cells (SGCs, which envelop the SGN cell bodies, allowing identification of individual SGNs; ref. 22) in mid-modiolar vibratome sections prepared from cochleae of WT and *Ndp-*KO. At 1 month, using a Tubb3 antibody to label SGNs and a Kir4.1 antibody to label SGCs (23), there was no difference between the 2 genotypes in the density (number/area) and size of SGNs in basal, middle, or apical cochlear regions (Figure 3, A–C). As anti-Tubb3 showed increased background staining of cochlear connective tissue in *Ndp*-KO mice at 11–12 months (due to immunoreactivity with the secondary antibody targeting mouse IgG; data not shown), we used immunostaining for the neuronal intermediate filament Nf200 (24) and myelin basic protein (Mbp) to quantify the density and size of SGN cell bodies at this stage (Figure 3, D–F). At 11–12 months old, there was a significant decrease in the density of SGNs only in the apical region of *Ndp*-KO mice (*P* = 0.028), not in the basal and middle regions (Figure 3E). These cells were significantly larger in all regions of the cochlea in *Ndp-*KO mice compared with WT mice (Figure 3F and Supplemental Figure 3). These data suggest that the ABR wave I abnormality detected at 1 month was not associated with a specific loss of SGNs. Although some pathological changes manifest, the SGNs survive in the middle and basal regions for at least 1 year.

### Degeneration of OHCs in Ndp-KO mice.

**Figure 4:**
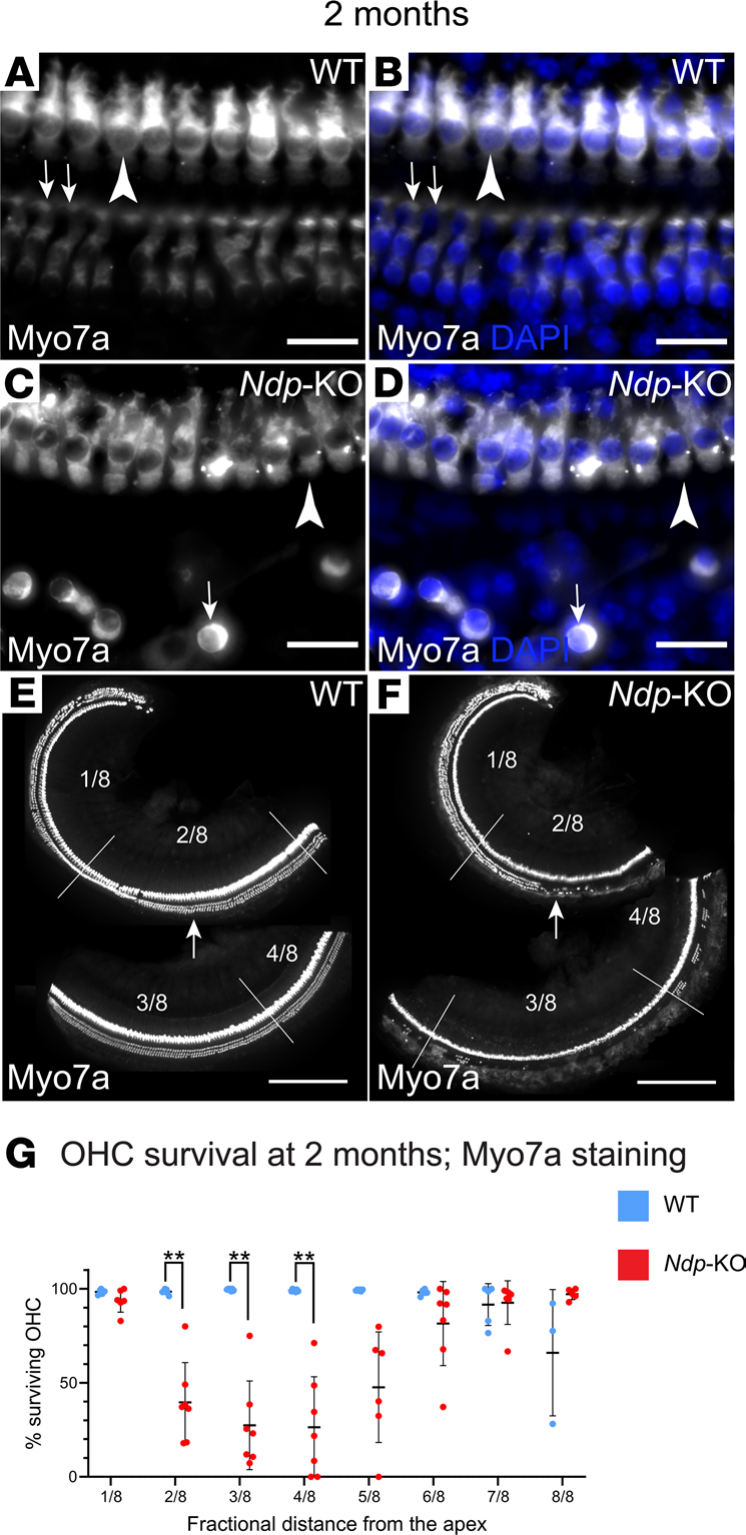
OHC loss in *Ndp-*KO cochlea. (**A**–**F**) Characteristic appearance of the organ of Corti of the WT and *Ndp-*KO at 2 months. White, anti–myosin VIIA (Myo7a) immunostaining; blue, DAPI. (**A** and **B**) In WT, IHCs (arrowheads) and OHCs (arrows) survive; example taken from middle region. (**C** and **D**) In *Ndp-*KO, IHCs survive (arrowheads), but the OHCs are degenerated in the middle region; arrows point to an example of a surviving OHC. (**E** and **F**) Low-magnification images of WT and *Ndp-*KO organ of Corti, showing the loss of OHCs; fractional distance from the apex is demarcated by lines; arrows point to comparable locations where they survive in WT (**E**). (**G**) Quantification of OHC loss across the length of the cochlea at 2 months (Myo7a staining). WT (blue) and *Ndp-*KO (red), mean ± SD, *n* = 7. Analysis with a mixed-model 2-way ANOVA and Šidák’s post hoc test indicated significant effects of both region (*P* < 0.0001) and genotype (*P* < 0.0001), and interaction of region and genotype (*P* < 0.0001). Šidák’s post hoc test indicated significant differences between comparable regions of WT and *Ndp-*KO in the apical regions at 2/8 to 4/8. *n* = 7 WT, *n* = 7 *Ndp-*KO analyzed; bars indicate mean ± SD. **P < 0.01. Regions of the organ of Corti defined as fractional distance from the apex (1/8 to 8/8): region 1/8 (3.1–6.1 kHz), region 2/8 (6.1–10.0 kHz), region 3/8 (10.0–15.0 kHz), region 4/8 (15.0–21.6 kHz), region 5/8 (21.6–30.2 kHz), region 6/8 (30.2–41.3 kHz), region 7/8 (41.3–55.9 kHz), region 8/8 (55.9–74.8 kHz). Scale bars: 20 μm (**A** and **B**), 250 μm (**C** and **D**).

We next investigated how the changes in auditory function were associated with hair cell survival. Full-length (apex–base) whole mounts of the organ of Corti were stained with phalloidin or with anti–myosin VIIA antibody and DAPI to delineate cell morphology and nuclei and to quantify hair cell degeneration (Figure 4, A–F, and data not shown). At 1 month, phalloidin staining showed that distribution of inner hair cells (IHCs) in *Ndp-*KO was similar to the WT with limited OHC loss observed in *Ndp*-KO (analyzed by a mixed-model 2-way ANOVA and Šidák’s post hoc test; fixed effect of genotype, *P* = 0.0241; fixed effect of region, *P* = 0.2508; fixed effect of region and genotype interaction, *P* = 0.2264). OHC loss was highest in the middle region at four-eighths of the length of the organ of Corti from apex to base, corresponding to approximately 16–21 kHz (91.79% ± 10.97%; mean ± SD OHC survival in region 4/8; adjusted *P* > 0.05, Šidák’s post hoc test; *n* = 6 WT, *n* = 7 *Ndp*-KO). By 2 months, substantial degeneration of the OHC had occurred, which distorted the morphology of the organ of Corti, hampering identification of individual viable OHCs by phalloidin staining, while the IHCs remained intact (data not shown). Labeling of hair cell bodies with anti–myosin VIIA antibody confirmed the integrity of IHCs in *Ndp-*KO at 2 months and allowed identification of surviving individual OHCs (Figure 4, A–F). We found that extensive OHC loss had progressed across a wide region corresponding to 6.1–21.6 kHz (two-eighths, three-eighths, and four-eighths of the length of the organ of Corti from apex to base; adjusted *P* = 0.0025, 0.0015, 0.0029, respectively, Šidák’s post hoc test) by 2 months in the *Ndp*-KO (mixed-model 2-way ANOVA; significant fixed effects of genotype, region, and genotype-region interaction, all *P* < 0.0001) (Figure 4G). OHC loss was thus most prominent in the apical half of the *Ndp*-KO cochlea. The apical part of the WT cochlea was not affected by degeneration, only some OHC loss was observed in the most basal region as is typical for C57BL/6 mice.

In summary, at 2 months, but not at 1 month, apical low-frequency functional deficits were detected by ABRs (6–18 kHz). Regional OHC loss and abnormally elevated DPOAE thresholds affecting a wider low- to mid-frequency range (up to 30 kHz) similarly progressed from near normal at 1 month to a marked pathology by 2 months. Considered together, these findings suggested that OHCs are initially functional and that degeneration may be secondary to an earlier insult. Our observation that EP was significantly lower than that of the WT at both 1 and 2 months indicated that there could be an early developmental malformation and dysfunction of the lateral wall cochlear tissue, which is responsible for generation and maintenance of EP.

### Cellular abnormalities of the cochlear lateral wall in Ndp-KO mice.

**Figure 5:**
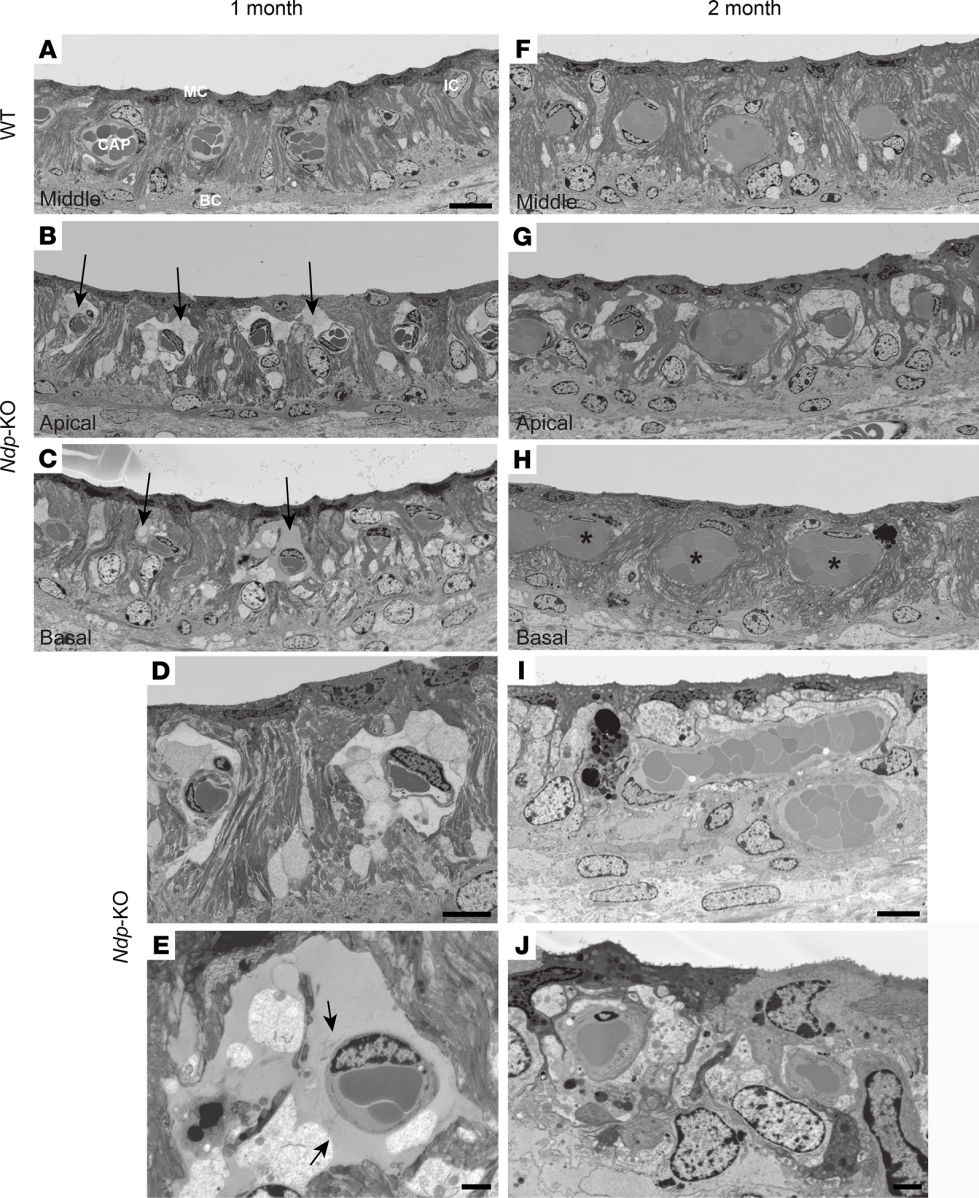
Electron microscopy of the cochlear lateral wall shows abnormalities of capillaries and marginal cells in *Ndp-*KO. Stria vascularis of 1-month-old WT (**A**) and *Ndp-*KO (**B**–**E**) mice and 2-month-old WT (**F**) and *Ndp-*KO (**G**–**J**) mice. (**A**) Middle turn of the cochlea in WT at 1 month shows normal architecture. MC, marginal cell; IC, intermediate cell; BC, basal cell; CAP, enclosed capillary. (**B**–**E**) In *Ndp-*KO at 1 month, large spaces surround the capillaries in apical coils (arrows in **B**) and extend to basal coils (arrows in **C**) in some animals. The capillaries are surrounded by shrunken and disrupted cells (**D**) and large extracellular spaces (**D** and **E**) that fill with material that stains with heavy metals to a density similar to that of plasma inside the capillaries (**E**). Loosely distributed fibrils (arrows in **E**) are present within the spaces close to the capillary. Most marginal cells are largely unaffected (**D**); they retain intense staining of the cytoplasm, appearing “dark,” and the extensive infoldings of the basolateral membrane with the characteristic elongated scalloped morphology of their nuclei located close beneath the luminal plasma membrane. **D** and **E** are higher magnifications of **B** and **C**, respectively. (**F**) Stria vascularis of WT at 2 months. (**G**–**J**) In *Ndp-*KO at 2 months, the capillaries are surrounded by intact cells filling the spaces apparent at 1 month (**G** and **H**). The stria appears thinner than at 1 month (**H** and **I**), and many capillaries appear enlarged (**H**; asterisks). (**I**) Marginal cells occupy a lower area, have lost their basal infoldings (**I** and **J**) and electron-dense staining, appear more rounded, and show changes in nuclear morphology (**J**). *n* = 6 WT, *n* = 6 *Ndp-*KO at 1 and 2 months. Scale bars: 10 μm (**A**, **D**, and **I**), 1 μm (**E**), 2 μm (**J**).

We analyzed the cochlear lateral wall by electron microscopy at 1 and 2 months to identify any abnormalities (Figure 5). The stria vascularis, a multilayered epithelium, lines the lateral wall, bounded by the scala media at its luminal surface and by the spiral ligament at its basal surface. A single layer of marginal cells extrudes K^+^ into the endolymph, producing the positive EP. The source of this K^+^ is the underlying syncytial cell layers, composed of intermediate cells, basal cells, and fibrocytes, which form a single syncytium due to the high density of gap junctions between them (25, 26). The main blood supply to the lateral wall is provided by 2 main capillary systems in the spiral ligament and in the stria vascularis with the latter network spreading between the marginal and intermediate cells supplying oxygen and nutrients to these cells (27).

By 1 month, significant abnormalities in *Ndp-*KO mice were apparent (Figure 5, A–E). The most marked feature at 1 month was the formation of large spaces around the capillaries in the stria vascularis (Figure 5, B and C, arrows). This was particularly prominent in the apical and into the middle turns but was also evident in the basal turns of some of the *Ndp-*KO animals. The spaces were filled by material that took up stain (Figure 5E), reminiscent of the light staining of blood plasma within capillaries (Supplemental Figure 4A), suggesting leakage of plasma from the capillaries. Fibrils within this electron density suggest disruption of the capillary basement membrane (Figure 5E and Supplemental Figure 4C). By 2 months, the large spaces around the capillaries had resolved (Figure 5, F–J), but some capillaries throughout the stria — apex to base — were enlarged (asterisks denote unusually large capillary cross sections); the mean cross-sectional areas were 50.6 μm^2^ ± 5.2 μm^2^ (SEM) in WT (50 capillaries from 6 animals) and 81.6 ± 10.5 μm^2^ in *Ndp*-KO (67 capillaries from 6 animals). The stria vascularis was also thinner; the mean distance from the luminal surface to the interface between basal cells and spiral ligament at 2 months was 32.4 ± 0.3 μm in WT (*n* = 22 measurements from 6 animals) and 29.4 ± 0.5 μm in *Ndp*-KO (*n* = 24 measurements from 6 animals). No differences in morphological features were identified after direct examination of endothelial tight junction structures from both WT and *Ndp*-KO (blind analysis was performed of 500 electron microscopy images of apical strial cross-section profiles of capillaries from 6 WT and 6 *Ndp*-KO animals at P30; 104 and 98 capillary profiles, respectively) (Supplemental Figure 4, G–L).

Some marginal cells also showed abnormalities: there was a loss of electron density; the extensive basolateral infoldings of the cell body were reduced, giving the cell a more smoothly rounded cell body; and the nucleus had changed shape at 1 month (Supplemental Figure 4E) and at 2 months (Figure 5, I and J, and Supplemental Figure 4F).

**Figure 6:**
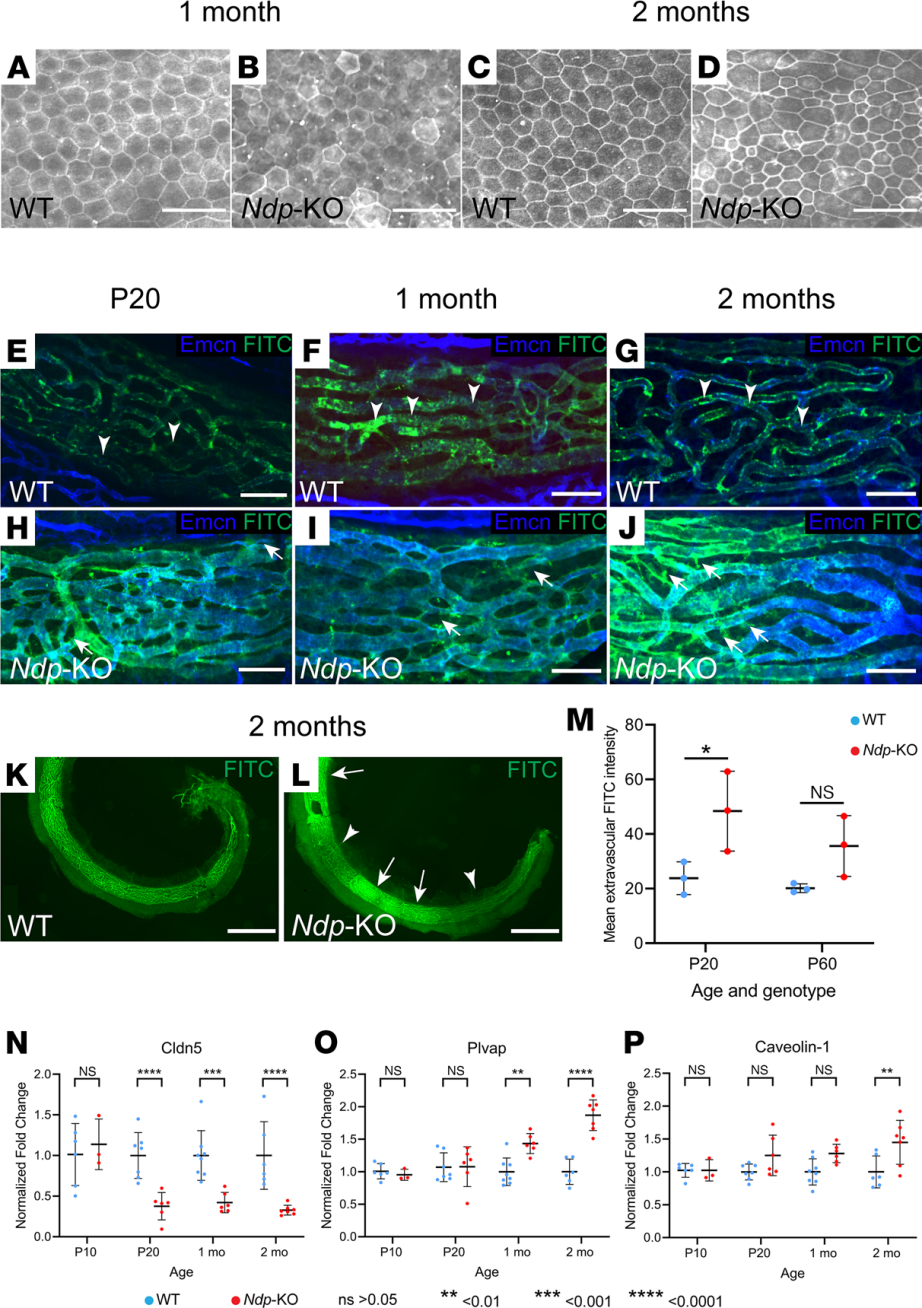
Marginal cell morphology and permeability of vascular barrier in *Ndp-*KO stria vascularis. (**A**–**D**) Anti-ZO1 immunostaining demonstrates evenly sized marginal cells at 1 month in the WT (**A**) and *Ndp*-KO (**B**) lateral wall; *n* = 3 WT, *n* = 4 *Ndp-*KO. At 2 months, WT marginal cells remain evenly sized (**C**), while those in *Ndp-*KO (**D**) are unevenly sized; *n* = 3 WT, *n* = 3 *Ndp-*KO. (**E**–**L**) Permeability assessments at P20 and 1 and 2 months using a fluorescent tracer assay (green, FITC-BSA; blue, vessels counterstained for endomucin). (**E**–**J**) FITC-BSA is restricted within the vessels (arrowheads) in WT, but detectable outside them in *Ndp*-KO (arrows). (**K** and **L**) At 2 months, FITC-BSA signal is uneven in *Ndp-*KO stria with patches of weak (arrowheads) and strong (arrows) intensity. Scale bars: 30 μm (**A**–**D**), 50 μm (**E**–**J**), 500 μm (**K** and **L**). (**M**) Extravascular FITC intensity is elevated in *Ndp*-KO compared with WT (2-way ANOVA; Šidák’s post hoc test showed *P* < 0.05 at P20; see also Supplemental Figure 5). Mean ± SD is represented; *n* = 3 WT, *n* = 3 *Ndp-*KO at 1 and 2 months. (**N**–**P**) Quantitative reverse transcriptase PCR analyses of vascular barrier and permeability marker gene expression. In *Ndp*-KO cochlea, *Cldn5* expression was significantly reduced from P20 (**N**), but *Plvap* and *Cav1* increased progressively from 1 and 2 months, respectively (**O** and **P**), compared with WT. Normalized fold change ± SD; 2-way ANOVA with Šidák’s post hoc test. *n* = 6 WT, *n* = 3 KO at P10; *n* = 7 WT, *n* = 6 KO at P20; *n* = 8 WT, *n* = 6 KO at 1 month; *n* = 6 WT, *n* = 7 KO at 2 months. *P ≤ 0.05, **P ≤ 0.01, ***P ≤ 0.001, ****P ≤ 0.0001.

To further investigate whether the marginal cells were affected by the lack of Ndp, we used anti-ZO1 staining, which delineates morphology of marginal cells by labeling their intercellular tight junctions. At 1 month, the shape and size of the marginal cells appeared similar between *Ndp-*KO and WT (Figure 6, A and B), but some cells stood out owing to a more defined ZO1 staining. By 2 months, morphological changes in *Ndp-*KO were apparent, as the size of the marginal cells became highly variable, with cells either enlarged or shrunken, indicating pathological change (Figure 6, C and D).

### Vessel permeability in the cochlear lateral wall in Ndp-KO mice.

As the electron microscopy analysis showed capillary abnormalities, we performed a functional evaluation of vascular barrier integrity in the *Ndp*-KO mice. Normal blood supply with an intact blood-labyrinth barrier between the cochlear lateral wall vasculature and the endolymph is critical for cells to maintain EP, and its disruption is a known mechanism of hearing loss. The blood-labyrinth barrier in the stria capillaries is composed of vascular endothelial cells, as well as pericytes and perivascular-resident macrophage-like melanocytes (28) (Supplemental Figure 4, A and B). We assessed the permeability of the capillary network to FITC-BSA (which has 66 kDa molecular weight and is an intermediate size tracer) at P20 and 1 and 2 months of age (Figure 6). Cochlear vasculature was counterstained for endomucin, which labels vascular endothelial cells. We found that the strial blood-labyrinth barrier was impermeable to FITC-BSA in WT mice (Figure 6, E–L) at all 3 time points, as evidenced by the colabeling of the stria vascularis vessels with endomucin antibody and FITC signal and a lack of FITC signal in the extravascular region of the lateral wall. By contrast, FITC signal was detected in extravascular regions of the stria in *Ndp-*KO mice at all time points (Figure 6, E–L). Quantification of the extravascular FITC-BSA signal in the stria vascularis (Figure 6M and Supplemental Figure 5) indicated the early onset of leakiness in the *Ndp*-KO, although FITC intensity varied among samples and along the length of the stria (Figure 6, K and L, and Supplemental Figure 5B), suggesting phenotypic variability. Thus, lateral wall vascular barrier breakdown precedes the anatomically distant OHC loss.

We conducted quantitative reverse transcriptase PCR analyses to compare differences in gene expression levels of the following markers that may affect vascular permeability between WT and KO cochlea at each time point (P20, 1 month, 2 months): *Cldn5*, a component of pericellular tight junctions between vascular endothelial cells (29); and *Plvap* and *Cav1*, involved in transcellular transport, through vessel fenestration and caveolae, respectively (30–32) (Figure 6, N–P). *Cldn5* was differentially expressed as early as P20. *Plvap* showed a higher expression in the *Ndp*-KO mouse cochlea from 1 month and *Cav1* from 2 months. These data support the idea that pericellular vascular permeability may be affected as early as P20 even though tight junctions appear normal at a morphological level (Supplemental Figure 4, G–L). Transcellular permeability may be altered as early as 1 month.

### Perinatal onset of cochlea lateral wall vascular abnormalities in Ndp-mutant mice.

**Figure 7:**
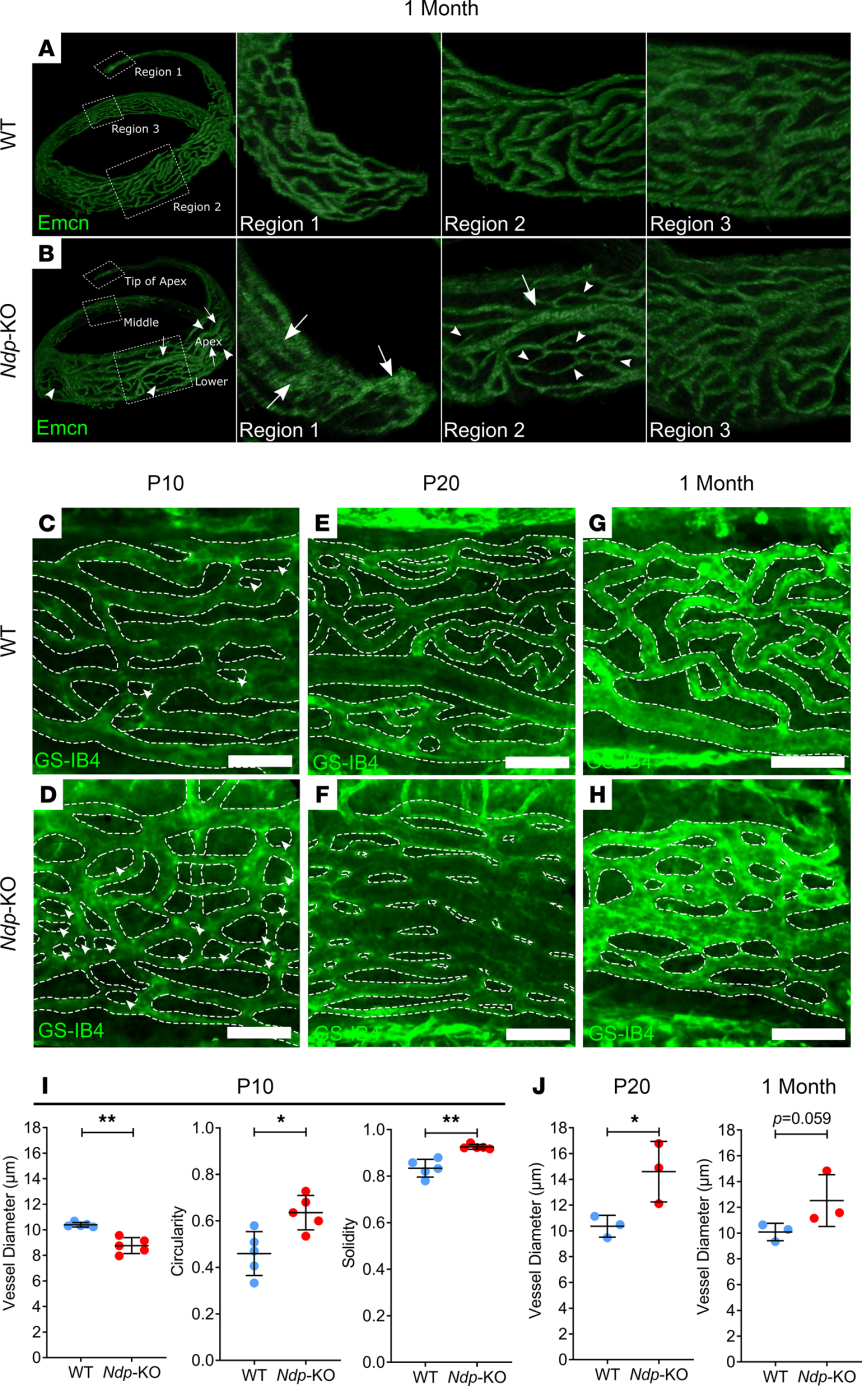
Postnatal onset of morphological abnormalities in stria vascularis capillaries in *Ndp-*KO. The stria vascularis of 1-month-old mice was examined with an antibody targeting endomucin. (**A** and **B**) Panels display the middle-apical region of the stria vascularis cropped from a 3D image of whole cochleae from WT (**A**) and *Ndp-*KO (**B**) mice. The regions indicated by perforated boxes in **A** and **B** are displayed in adjacent panels. White arrows indicate dilated capillaries, white arrowheads indicate narrow capillaries and regions with sparse vessels. (**C**–**H**) The apical region of the stria vascularis was isolated and examined with GS-IB4. WT and *Ndp-*KO were examined at P10 (**C** and **D**), P20 (**E** and **F**), and 1 month (**G** and **H**). White arrowheads indicate intercapillary regions that are small and circular/oval in structure. (**I**) Quantification of vessel diameter and shape descriptor measurements of intercapillary regions for P10 stria vascularis; *n* = 5 WT, *n* = 5 *Ndp-*KO; bars indicate mean ± SD. (**J**) Quantification of vessel diameter in P20 and 1 month stria vascularis; *n* = 3 WT, *n* = 3 *Ndp-*KO; bars indicate mean ± SD. **I** (circularity) and **J** analyzed with an unpaired *t* test, **I** (vessel diameter and solidity) analyzed with Mann-Whitney test; *P < 0.05, **P < 0.01. Scale bars: 50 μm.

Development of the microvessels of the stria vascularis and spiral ligament occurs progressively from the basal turn in postnatal mice, completing by P8 (33), which corresponds to the age at which a rise in EP can be first measured (21, 34). As EP did not achieve maximal values in the *Ndp*-KO and vessel integrity was compromised in juvenile and adult mice, we reasoned that the postnatal stages of cochlear microvasculature development may be impaired in the absence of NDP. We examined the morphology of the stria vasculature during the first postnatal month in cochlear whole-mount preparations by staining endothelial cells with endomucin antibody. At 1 month, endomucin immunostains revealed clear qualitative differences between the morphology of stria capillaries in the apical cochlear turns of WT mice and *Ndp-*KO mice (Figure 7, A and B, regions 1 and 2). The peculiarities in *Ndp-*KO mice were more evident in the cochlear apical region than in the more basal regions. In the middle turn, the vessels of *Ndp-*KO mice appeared comparable to those of WT mice (Figure 7, A and B, region 3). In WT mice, vessels appeared uniform in diameter and evenly distributed across the capillary net (Figure 7A). In contrast, in *Ndp-*KO mice the vessels were less defined, appearing more variable in diameter (Figure 7B, arrows and arrowheads). See also videos of 3D rotating image (Supplemental Videos 1 and 2).

Next, the apical stria vascularis was isolated and laid flat for imaging of GS-IB4 fluorescence, in order to define the vessel morphologies more clearly and to quantify the apparent differences between genotypes. The vessels of the apical stria vascularis of P10, P20, and 1-month-old *Ndp-*KO mice showed a marked difference in morphology compared with those of WT mice (Figure 7, C–H). One-month *Ndp-*KO vessels had more variable thickness and circular intercapillary regions in comparison with WT mice (Figure 7, G and H). This variation was also apparent at P20 (Figure 7, E and F) but less so at P10 (Figure 7, C and D). In P20 *Ndp-*KO mice there were areas of weak contrast between capillary and intercapillary regions, particularly where the phenotype was pronounced. However, at P10 when the phenotype was not as severe, it was easier to delineate vessel boundaries, and there was a decrease in the average diameter of vessels in *Ndp-*KO mice (Figure 7I). Additionally, the intercapillary regions were more circular and more solid (i.e., regular/even boundary) (Figure 7I). At P20, the analysis revealed a significant quantitative difference between the mean vessel diameters in the 2 genotypes (Figure 7J). At 1 month, there was no significant difference, but the measurements were complicated by variabilities in the severity of the phenotype in *Ndp-*KO mice. Together, the data suggest that the vessels of stria vascularis do not develop normally, and then show progressively disordered organization.

### Abnormal pericyte coverage and disruption of tight junction proteins in the lateral wall.

To examine how these early capillary structural malformations lead to vessel leakiness, we investigated the arrangement of perivascular cells and the distribution of endothelial cell tight junctions during development. Vascular endothelial cells connected by tight junctions line the capillaries (Supplemental Figure 4, A and B) and are surrounded by a basement membrane in which pericytes lie embedded and regulate capillary diameter, also playing a role in regulating blood flow, vascular permeability, and maintenance of microvessel wall integrity. The distribution, density, and morphology of pericytes are known to be region specific (approximately 1:2 ratio of endothelial cells to pericytes in the lateral wall) and are tightly coupled to metabolic demand (27, 35). Pericytes on the capillaries of the stria vascularis and the spiral ligament are normally rich in the intermediate filament protein desmin, giving mechanical strength and integrity of the network (27). Abnormal pericyte coverage was previously observed in the retinal vasculature of *Ndp-*KO mice (36, 37), and after endothelial cell–specific conditional knockout of *Sox17*, a known target of Ndp (16, 38). Reduced pericyte numbers have also been reported in the cochlea of a mouse model of Alport syndrome (39), in which hearing loss is associated with strial dysfunction.

**Figure 8:**
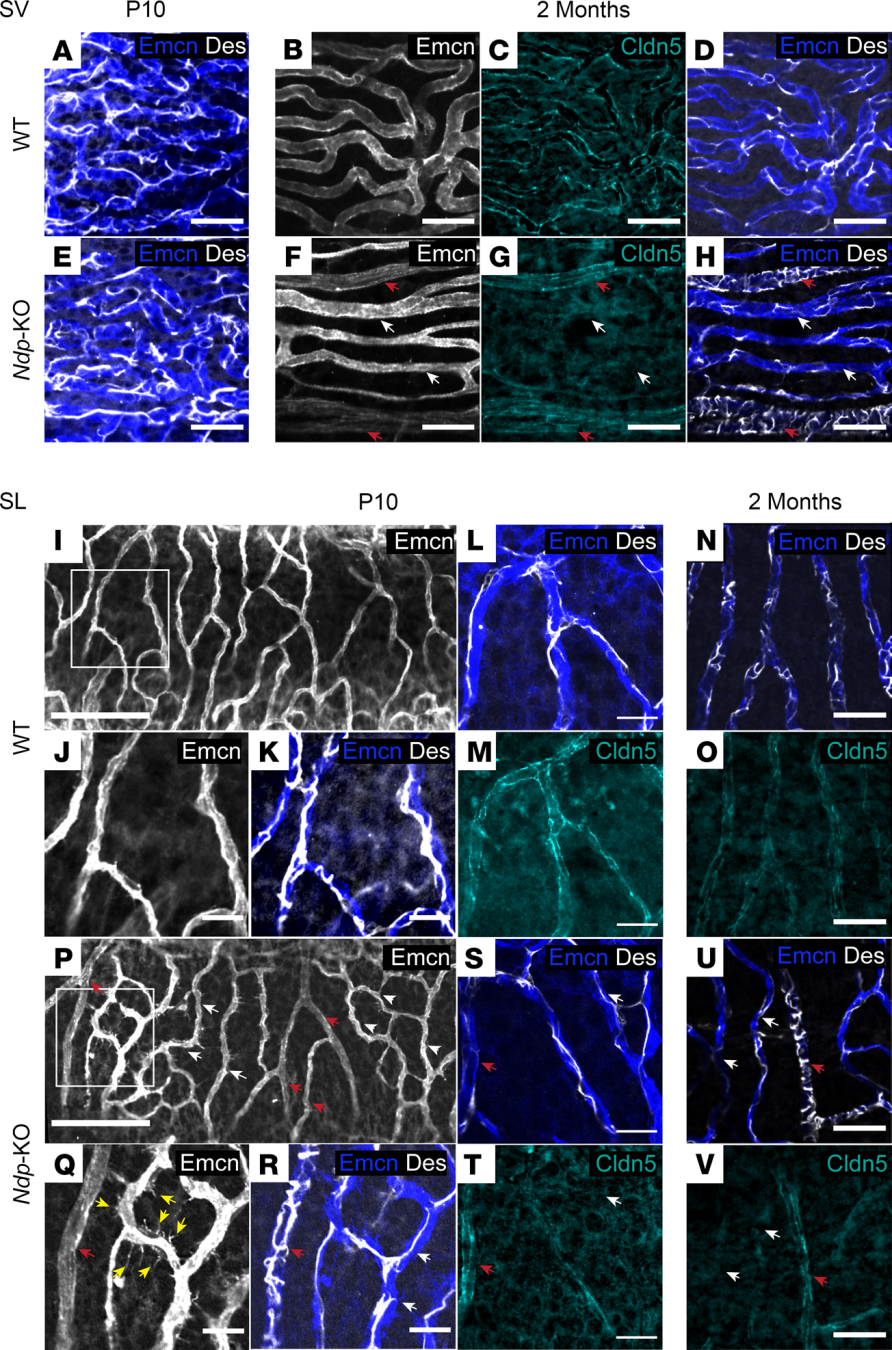
Abnormal pericyte coverage of capillaries in the lateral wall affecting the spiral ligament and stria vascularis. (**A**–**V**) Stria vascularis (SV) (**A**–**H**) and spiral ligament (SL) capillaries (**I**–**V**), immunostained with vessel marker anti-endomucin (Emcn), pericyte marker anti-desmin (Des), and vascular tight junction marker anti–claudin-5 (Cldn5) antibodies. (**A** and **E**) At P10, pericytes (Des, white) are evenly distributed across the SV (Emcn, blue) in both WT (**A**) and *Ndp-*KO (**E**). (**B**–**H**) At 2 months, *Ndp*-KO strial capillaries (**F**–**H**) are either high-endomucin, low–claudin-5, low-pericyte-coverage vessels (white arrows, **F**–**H**) or low-endomucin, high–claudin-5, dense-pericyte-coverage vessels (red arrows, **F**–**H**), compared with the even staining patterns of WT strial capillaries (**B**–**D**). Scale bars in **A**–**H**: 30 μm. (**I**–**O**) At P10 and 2 months, WT SL capillaries (**I**–**K**) form regular branches (Emcn, **I**, **J**, and **N**) with even pericyte coverage (desmin, **K**, **L**, and **N**; white) and claudin-5 staining (**M**; cyan). **J** and **K** are a higher magnification of **I**. (**P**–**V**) In contrast, *Ndp-*KO SL capillaries are abnormal from P10, showing either irregular patterns of high-endomucin-staining meshworks (**P**, white arrows) with filopodia (**Q**, yellow arrows) and low pericyte coverage (**R**: Des, white; white arrows), or, alternatively, low endomucin staining (**P**–**T**, red arrows) and abnormally dense pericyte wrapping (**R** and **U**: Des, white; red arrows). **Q** and **R** are a higher magnification of **P**. Like in the SV, abnormal vessels with high pericyte coverage show claudin-5 (**V**, red arrow), whereas vessels loosely covered with pericytes show low or absent claudin-5 (**V**, white arrows), compared with the pattern in WT (**N** and **O**). Scale bars in **I**–**V**: 100 μm (**I** and **P**), 20 μm (all others). *n* = 9 WT, *n* = 8 *Ndp-*KO analyzed at P10, and *n* = 14 WT, *n* = 15 *Ndp-*KO analyzed at 2 months, for desmin/endomucin/claudin-5 costaining.

Claudin-5 (CLDN5), a critical component of endothelial tight junctions that control pericellular permeability, is also affected in *Ndp-*KO retina and brain vessels (13, 40). We performed immunostaining for desmin and claudin-5, together with endomucin labeling of the endothelial cells (Figure 8). At P10, pericyte coverage of the capillary network of the stria vascularis appeared comparable between the WT and *Ndp-*KO (Figure 8, A and E). By 2 months, the capillary networks of the WT stria vascularis maintained an even coverage of desmin-labeled pericytes and showed claudin-5 labeling (Figure 8, B–D). By contrast, *Ndp-*KO vessels had abnormally diversified into either large vessels, with low-endomucin, dense transversely wrapped pericytes and high claudin-5, or vessels with high-endomucin, sparse pericyte coverage and loss of claudin-5 expression (Figure 8, F–H).

We found that this atypical capillary phenotype extended to the spiral ligament capillary network. Spiral ligament vessels normally have an even pericyte coverage (Figure 8, I–K) with clear claudin-5 labeling by P10 (Figure 8M). However, by this stage *Ndp-*KO spiral ligament capillaries already displayed abnormal diversification into more densely or sparsely pericyte-covered vessels (Figure 8R), a feature that was maintained at 1 month (data not shown) and 2 months (Figure 8U). In addition to the atypical pericyte coverage in the spiral ligament capillaries, the thin *Ndp-*KO vessels had high numbers of filopodia and formed meshworks between themselves (Figure 8, P and Q). The atypical pericyte coverage extended to the 2-month time point, but abnormal filopodia were less apparent by this stage (Figure 8, U and V).

Claudin-5 protein distribution was abnormal in spiral ligament capillaries of *Ndp-*KO mice as early as P10 (Figure 8, S and T) compared with WT (Figure 8, L and M) with claudin-positive (high signal) and -negative (low signal) vessels. By 2 months, *Ndp*-KO mice continued to show a distinctive abnormal claudin-5 distribution in the ligament compared with WT (Figure 8, N, O, U, and V). Like in the stria, this abnormal distribution was associated with the altered pericyte coverage. The vessels that had a dense pericyte wrapping pattern expressed claudin-5 in both stria and ligament (Figure 8, F–H, U, and V).

We quantified pericyte coverage of strial and spiral ligament capillaries, in *Z*-stack images of lateral wall whole mounts labeled for endomucin (endothelial cells) and desmin (pericytes). The total percentage area of vessels covered by pericytes was compared between *Ndp*-KO and WT (Supplemental Figure 6). Reduced pericyte coverage was found in both the stria vascularis and the spiral ligament vessels in the *Ndp*-KO (WT 18.43% vs. *Ndp*-KO 14.92%, *P* = 0.036, in the stria; WT 29.96% vs. *Ndp*-KO 20.08%, *P* = 0.0371, in the spiral ligament) (Supplemental Figure 6, C and E). In the spiral ligament, the high-endomucin capillaries had the lowest coverage of 18.42% (Supplemental Figure 6F) compared with WT.

Together these data demonstrate an early abnormal organization of pericytes within the developing cochlear microvasculature and progressive remodeling and disruption of tight junctions, which are likely to cause the later pathologies.

## Discussion

The main goals of this study were to define the timing of the onset of hearing loss in Norrie disease, and to confirm the site(s) of pathological lesions occurring within the auditory system of the *Ndp-*KO model of Norrie disease. Based on our observations of a strial defect and reduced EPs as early features, the hearing loss in Norrie disease is probably best classed as a strial or metabolic defect (41), which leads on to neural defects. This information increases our understanding of the cellular basis of this debilitating disease and may also help in the design of therapeutic approaches aimed at preventing hearing loss in patients.

Our review of hearing loss in a small set of patients with Norrie disease showed in all but 1 child that the hearing loss was mild to moderate at onset, but in a single 5-year-old, a moderate to profound hearing loss was detected. This indicates that in some children, the progression of hearing loss can be accelerated. The hearing loss affected low or high frequencies first in different individuals, and there was no indication of OAE abnormality in 1 child in whom this test was done when hearing loss was mild. Over time the hearing loss in the patients with Norrie disease progressed to be bilateral and flattish. Hearing loss was documented in all children before the age of 11 years, and as early as 3 years of age, where audiograms were available. There are several previous reports suggesting that a flattish audiogram shape is likely to indicate a strial defect, and it is interesting that the audiograms of these 6 Norrie patients have flattish shapes (or at least not the typical steep downward slope at high frequencies that is often seen, which is more likely due to hair cell defects) (42, 43).

Our analysis of the Norrie disease mouse mutant extends previous observations (10), and highlights the surprisingly early onset of the vascular phenotype, rapid advance of pathology in the cochlear lateral wall, and a loss of hearing sensitivity in mice as early as 1 month of age progressing to significant impairment and auditory threshold increases across a range of low to mid-frequencies (6–18 kHz) by 2 months. Our study analyzed *Ndp* loss of function in the largely C57BL/6 genetic background, whereas the earlier study analyzed the effect on a more mixed background (C57BL/6, 129/Sv, CD1) outcrossed for 2 generations to CBA/CaJ and found impairments at all different frequencies at 3–4 months progressing to relatively flat profound loss by 15 months (10). Notably, this mixed-genetic-background study described exceptions where 1 Norrie disease mouse showed substantial hearing loss at 3 months and 1 retained hearing in the middle frequencies at 13 months. Together these findings suggest that genetic modifying factors affect timing and site of disease onset and progression.

### Pathology in cochlear microvasculature of Ndp-KO mice progresses from hearing onset.

We found that the hearing loss in the *Ndp*-KO mouse follows early developmental malformation of the cochlear microvasculature and a progressive impairment of stria vascularis function, resulting in a reduced EP. Our analysis of the microanatomy of the cochlea shows that malformation of the cochlear vasculature in the postnatal period, of both the stria vascularis and the spiral ligament, precedes functional impairment of OHC and the death of these cells in young adult animals. The sensory hair cells, like the neurons of the brain, are known to be vulnerable to ischemic injury (44, 45). Blood flow in the strial capillaries is nonpulsatile and slower than in the vessels of the spiral ligament that have a primary role in regulating blood flow to the lateral wall. Strial capillaries instead play a crucial role in maintaining EP, ion transport, and endolymph fluid balance needed for hearing sensitivity (46). Malformation of both of these connected cochlear capillary networks is indicated as a primary lesion site in the auditory system in Norrie disease. A likely contributor to the fluctuating hearing loss in Norrie disease is the early degeneration and remodeling of the malformed stria vascularis, which plays a critical role in the generation of the high-potassium endolymph, and maintenance of the EP that acts as a “battery” for hair cell transduction during normal hearing (47). A postmortem temporal bone study of a 77-year-old man diagnosed with Norrie disease revealed marked atrophy of stria vascularis, beyond that explainable by normal aging (6). Various progressive cochlear defects have previously been described in aging *Ndp*-mutant mice (10). In that study, blood vessel enlargement was evident from 2 months, and cellular loss of hair cells, spiral ganglion cells, and stria vascularis from 3–15 months. In the present study, morphological abnormalities were evident in the cochlear microvasculature by P10. This abnormality in the cochlear apex occurs around hearing onset and develops further as hearing matures.

These studies indicate that NDP is needed for normal postnatal development and organization of the cochlear microvasculature with subsequent deterioration involving remodeling and loss of tight junction integrity. The specificity of vessel abnormalities to the cochlear apical region may provide a clue to the disease progression. For example, this may be due to specific hemodynamic demands in the apical stria vascularis or the fact that developmental maturation is incomplete as division of the single-layer capillary at birth into the microvessels of the stria and spiral ligament normally occurs from the base to the apex (33). The early abnormal organization of pericytes surrounding the vessels also appears to play a role. Pericytes may respond deleteriously to loss of Norrin protein, impacting cell proliferation and survival via changes within the WNT pathway (36, 37). Pericytes regulate capillary diameter (48), and perivascular-resident macrophage-like melanocytes are known to regulate the intrastrial fluid–blood barrier (28). Both play a role in angiogenesis and vascular stability in the cochlea (49).

### Early-onset hearing loss precedes loss of primary afferent neurons.

Before this study, hearing loss in *Ndp*-KO mice has been reported in aging mice from 3 to 15 months (10). However, abnormalities in stria and spiral ligament microvasculature were detected here as early as P10. Hearing onset occurs in mice around P14, shortly after the vascular maturation (33). We found effects on the neural activity within the cochlea by 1–2 months in *Ndp*-KO mice, suggesting that the pathological events are already under way at this age. Our observations suggest that hearing ability is near normal in *Ndp*-KO juvenile mice aged 1 month. At 1 month, OHC damage was first apparent but we did not identify a loss of SGNs, which largely survived intact in aged mice. By 11–12 months, limited loss of SGN numbers in *Ndp*-KO mice was restricted to the apical cochlear region, suggesting a local effect only. There was also an increase in the size of SGN cell bodies in all regions in *Ndp*-KO mice, a phenotype that may be a consequence of changes in homeostasis of the extracellular environment within the ganglion.

### Ndp-mutant mice as an effective platform for therapy development.

*Ndp*-KO mice have attributes that model juvenile-onset hearing loss in Norrie disease and therefore are useful for therapy development. Examination of stria vascularis and spiral ligament abnormalities provides a means to examine the direct effectiveness of therapeutic interventions at a cellular level as early as P10. Similarly, ABR recordings in young adult mice could be an effective assay of intervention success, to judge whether the early-onset hearing loss is still evident by 2 months. The opportunity to test therapeutic interventions early is useful, as it allows investigation of the efficacy of therapeutic interventions in a relatively quick and cost-effective way.

Our study on the *Ndp*-KO model indicates that early malformations of the cochlear microvasculature are initially compatible with normal development and function of the organ of Corti, enabling hearing within normal range. Therapies to prevent deterioration of vessel integrity leading to vessel leakiness and promote normal blood flow, and/or protect hair cells, may be of value for treating Norrie disease hearing loss. For example, regulating levels of claudin-5 has been shown to abrogate disease symptoms in other models of neurodegenerative disorders (50).

It is also important to consider the target and timing of therapies, whereby small molecules that correct perturbed signaling pathways, or gene therapy to deliver healthy expression of NDP, are promising approaches. The best targets will likely be those cells that normally express NDP, for example, Müller glia in the retina (16) and astroglia in the brain (51). The early ectopic expression of *Ndp* and intravitreal injection of NDP in the eye can negate retinal pathology in *Ndp*-KO mice (52, 53), and similar approaches may ameliorate the ear pathology. While there is clear evidence that *Ndp* is expressed in stria vascularis (54), the precise cells expressing *Ndp* are yet to be identified. We performed an analysis of recently published single-cell RNA-Seq raw data from adult mouse cochlear lateral wall and found evidence of higher *Ndp* expression within basal cells (cluster expressing *Cldn11*) compared with other cell types in the lateral wall (55) (Gene Expression Omnibus database, GSM4618125/GSM4618124; Supplemental Figure 7). Future characterization of strial cells that secrete Ndp is a priority as well as the development of approaches that target those cells. Timing of intervention will also be critical to determine whether gene replacement approaches can prevent disease progression despite the early developmental pathology. It is possible that systemic delivery of therapeutic agents will be more successful allowing targeting of multiple types of cells for most effective treatment.

### Implications for understanding the progression of hearing loss in Norrie disease.

This study reports observations from mice with a knockout mutation in the *Ndp* gene. While we must be cautious when making direct comparisons between mice and humans, these findings contribute to understanding the onset and progression of the disease in patients. Some patients with Norrie disease present hearing loss from early childhood (as reported here and by others; ref. 5). It is difficult to investigate the condition of the cochlear microvasculature in patients, so it is unclear when this tissue is perturbed in humans. Given the results of this study, it is reasonable to suspect that the pathologies leading to hearing loss are already under way before its detection in the clinic. Therefore, it may be useful to monitor hearing ability from birth using OAEs and ABRs or to measure cochlear blood flow, as this may provide important information about perturbed activity and potential hallmarks of disease onset and progression. This will provide much-needed information about the progression of Norrie disease and a more accurate prognosis for patients.

In some cases, patients with Norrie disease have received cochlear implants to help overcome their dual sensory loss (5). Understanding the biological causes of hearing loss and its onset and progression will have important implications for the monitoring of hearing, use of OAE and ABR, timing of cochlear implants, and prediction of the outcome of such a treatment. For example, most SGNs appeared to survive the vascular pathologies in *Ndp*-KO mice. If this is the case in humans, early electrical stimulation of the auditory nerve via cochlear implants may protect against the degeneration of SGNs, and consequently favor an improved outcome. Further investigations must be carried out to better define the onset, progression, and nature of hearing loss in Norrie disease. In conjunction with preclinical animal studies, we might then design effective therapies for the prevention of dual sensory loss in Norrie disease.

## Methods

Detailed methods are provided in Supplemental Methods.

### Study approval.

Informed consent was obtained from parents or guardians of patients prior to participation. The study was approved by the National Research Ethics Committee London–Queen Square (identifier 17/LO/0841; Understanding the clinical features in patients with Norrie Disease). Animal studies were carried out after University College London and King’s College London Ethics Review approval and in accordance with UK Home Office regulations and the UK Animals (Scientific Procedures) Act of 1986 under UK Home Office license.

## Author contributions

The study was conceptualized by MBG, JCS, WB, VP, DB, and AF. Investigation was carried out by VP, DB, NJI, LAA, and AF. Methodology was developed by VP, DB, NJI, AP, KES, DAM, DJ Jafree, DAL, AAY, DJ Jagger, AF, and JCS. Data analysis and interpretation were done VP, DB, NJI, AP, WP, KES, YCL, KPS, DJ Jagger, AF, WB, and JCS. Resources and funding were acquired by MBG, JCS, and WB. The manuscript was written by VP, DB, NJI, AP, AF, and JCS. All authors provided critical feedback and helped shape the research, analysis, and manuscript. The final manuscript was approved by JCS.

## Supplementary Material

Supplemental data

Supplemental video 1

Supplemental video 2

## Version 1 02/08/2022

Electronic publication
