# Supplementary material for: The timing of auditory sensory deficits in Norrie disease has implications for therapeutic intervention
Source: JCI Insight. 2022 Feb 8;7(3):e148586. doi: 10.1172/jci.insight.148586 (PMC8855802; doi:10.1172/jci.insight.148586)
Supplement: Supplemental data [file jciinsight-7-148586-s188.pdf]

SUPPLEMENTAL DATA

The Timing of Auditory Sensory Deficits in Norrie Disease has Implications for Therapeutic Intervention

D. Bryant*, V. Pauzuolyte*, N. J. Ingham, A. Patel, W. Pagarkar, L. Anderson, K. E. Smith, D. Moulding, Y.C. Leong, D. Jafree, D. Long, A. Al-Yassin, K. P. Steel, D.J. Jagger, A. Forge, W. Berger, J. C. Sowden◆, M. Bitner-Glindzicz

Supplementary Data / Video 1: 3D rotation video (.avi file) of whole mount apical 1 month WT stria vascularis. Rotation of whole mount cochlear stained with endomucin. The stria vascularis was computationally dissected (by cropping each z-slice) to observe it alone.

Supplementary Data / Video 2: 3D rotation video (.avi file) of whole mount apical 1 month *Ndp*-KO stria vascularis. Rotation of whole mount cochlear stained with endomucin. The stria vascularis was computationally dissected (by cropping each z-slice) to observe it alone.

Supplementary Data Figures 1-7

Supplementary Methods

Supplementary Table 1

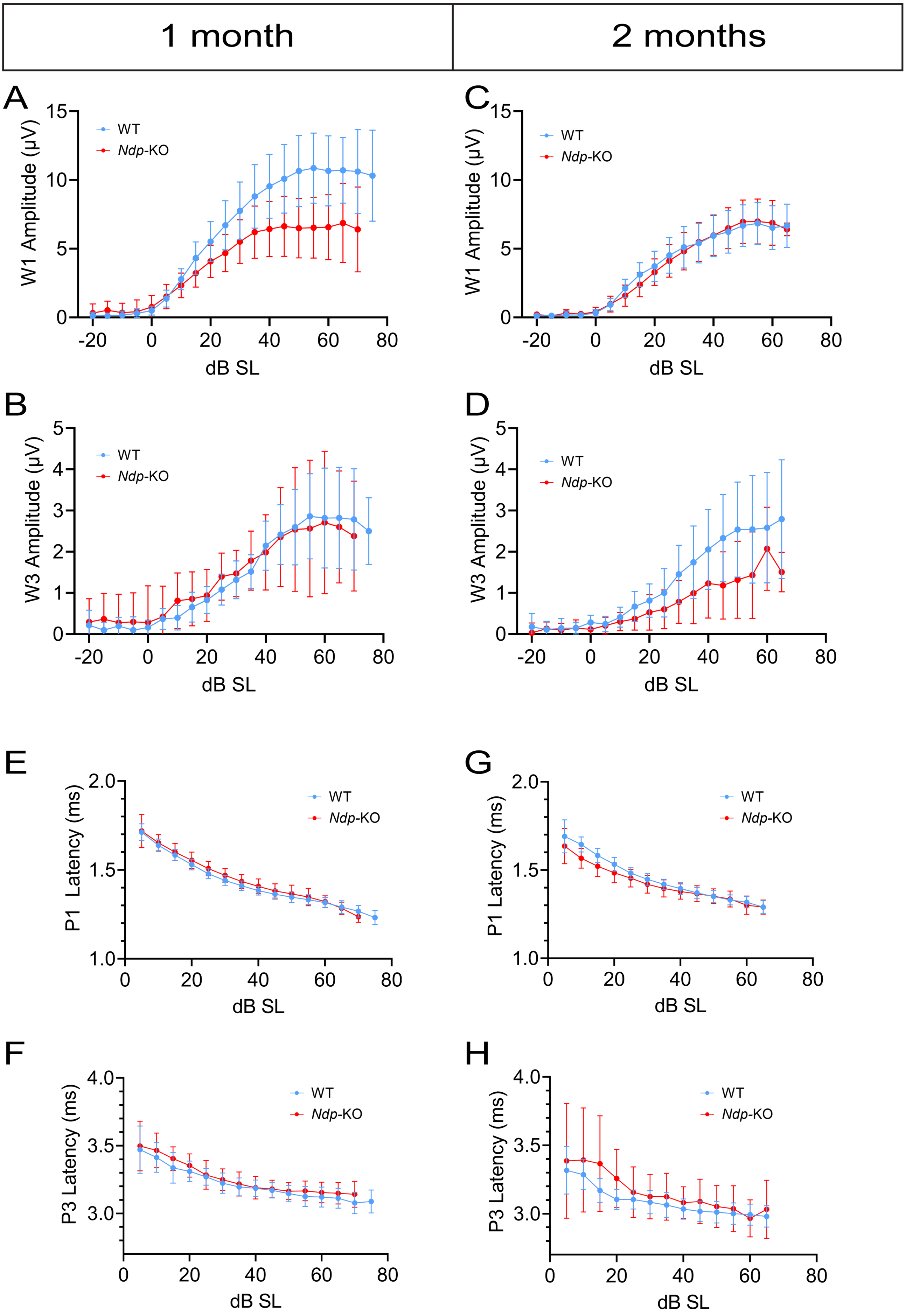
1 month
2 months
A
B
C
D
E
F
G
H
W1 Amplitude (μV)
W3 Amplitude (μV)
P1 Latency (ms)
P3 Latency (ms)
dB SL
WT
Ndp-KO

Supplementary Figure 1. Input/Output functions for click evoked ABRs in *Ndp*-KO mice.
A-D ABR wave 1 (**A, C**) & wave 3 (**B, D**) peak-to-peak amplitude (mean ± SD) is plotted as function of dB sensation level (SL, dB relative to threshold) for WT mice (blue) and *Ndp*-KO mice (red) aged 1 month (**A, B**) and 2 months (**C, D**). **E-H.** ABR wave Peak 1 (**E, G**) latency & Peak 3 (**F, H**) latency (mean ± SD) is plotted as function of dB sensation level (SL) for WT mice (blue) and *Ndp*-KO mice (red) aged 1 month (**E, F**) and 2 months (**G, H**). N = 11 WT, N = 12 *Ndp*-KO analysed at 1 month, N = 9 WT, N = 13 *Ndp*-KO analysed at 2 months.

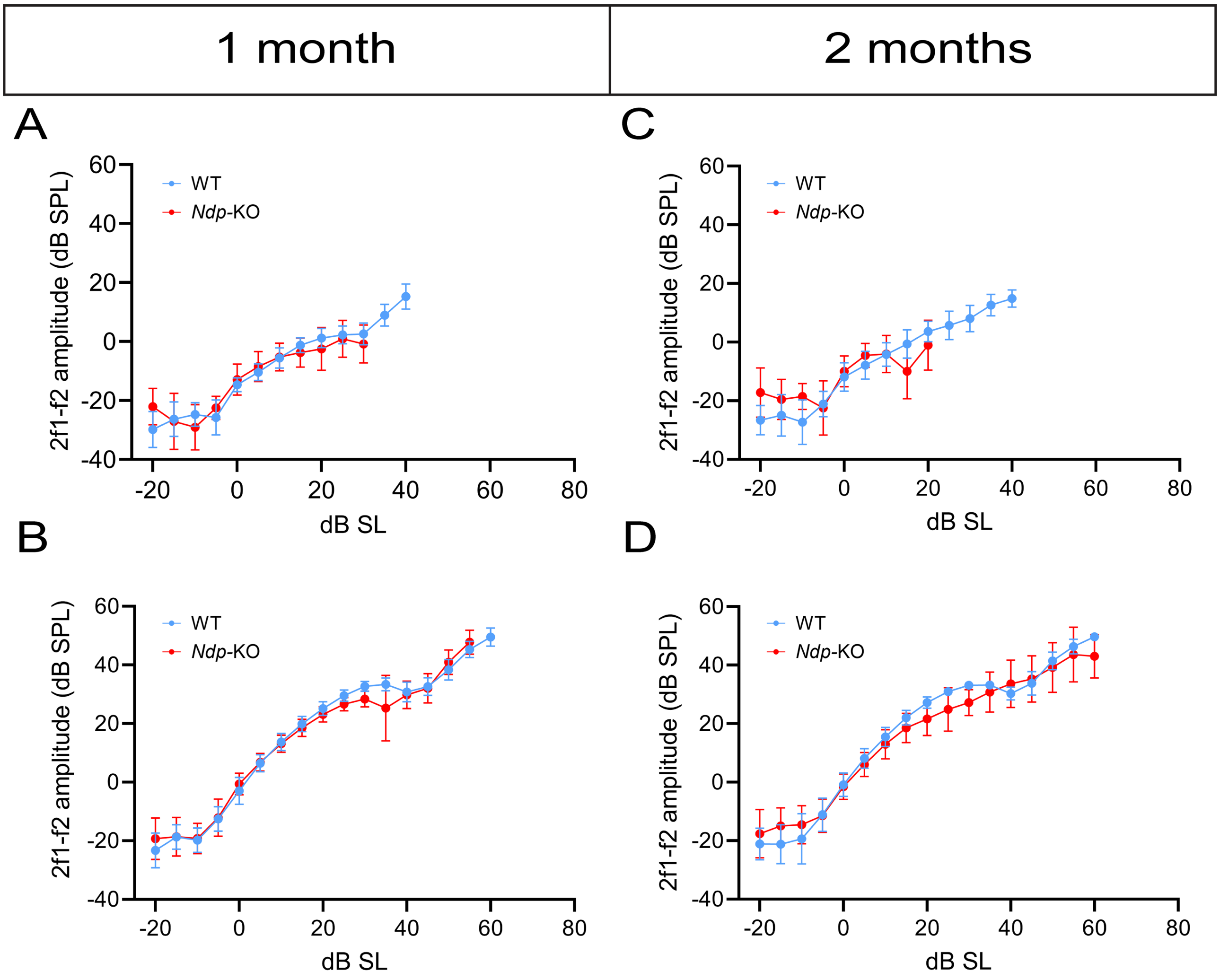

Supplementary Figure 2. Input/Output functions for DPOAEs in *Ndp*-KO mice.

A-D Examples of growth functions of the 2f1-f2 DPOAE for 6 kHz (**A, C**) and 18 kHz (**B, D**) f2 frequencies. DPOAE amplitude (mean ± SD) is plotted as function of dB sensation level (SL, dB relative to threshold) for WT mice (blue) and *Ndp*-KO mice (red) aged 1 month (**A, B**) and 2 months (**C, D**). N = 10 WT, N = 12 KO analysed at 1 month, N = 9 WT, N = 13 *Ndp*-KO analysed at 2 months.

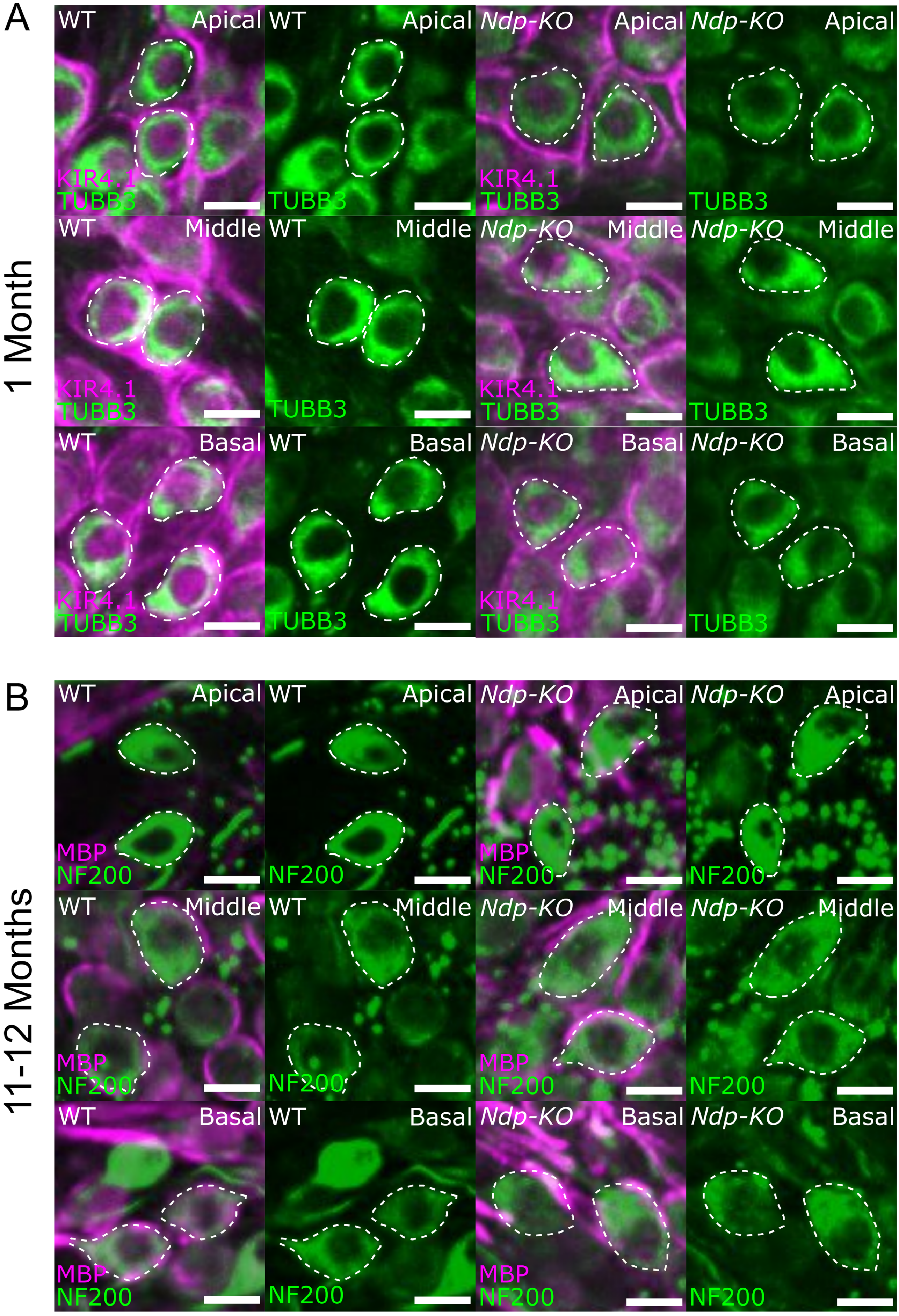

A
B
1 Month
11-12 Months
WT
Ndp-KO
Apical
Middle
Basal
KIR4.1
TUBB3
MBP
NF200

Supplementary Figure 3. Enlarged spiral ganglion neurons.

A Apical to basal spiral ganglia of WT and *Ndp-KO* mice at 1 month showing TUBB3 is expressed in the spiral ganglion neurons and Kir4.1 is expressed in the surrounding satellite glial cells.
B Apical to basal spiral ganglia of WT and *Ndp-KO* mice at 11-12 months showing NF200 is expressed in the spiral ganglion neurons and MBP is expressed in the surrounding satellite glial cells. Scale bars = 10µm.

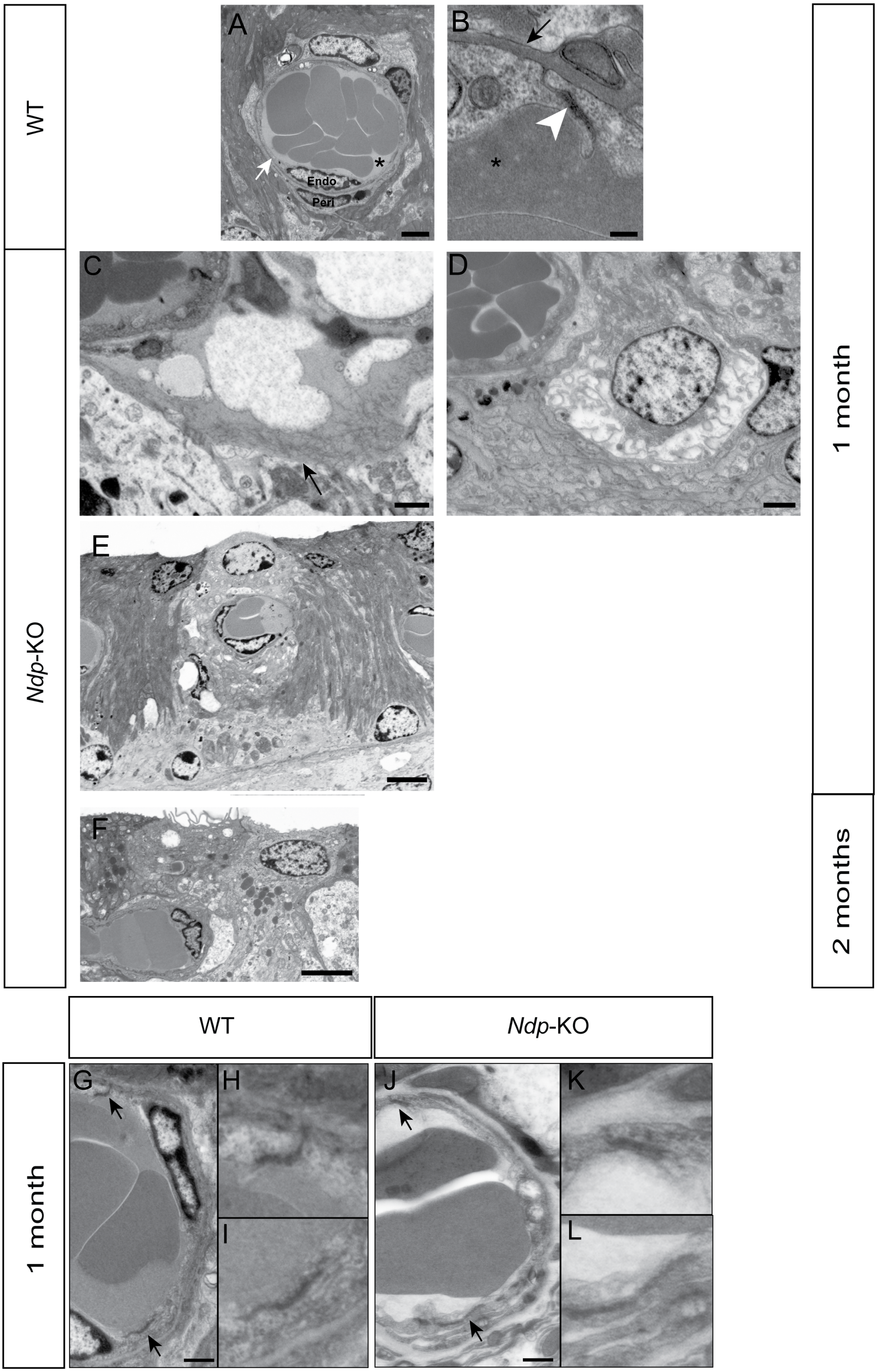

WT
Ndp-KO
1 month
2 months
A
B
C
D
E
F
G
H
I
J
K
L
Endo
Peri

Supplementary Figure 4. Electron microscopy of the cochlear lateral wall.

A, B EM showing normal strial capillary morphology in WT animals. The capillary lumen is enclosed by endothelial cells (endo in panel A) which are surrounded by a thin basement membrane (indicated by arrows in panels A and B) to which pericytes (peri in panel A) are closely apposed on the outer surface. The intercellular spaces between adjacent endothelial cells are sealed with tight junction (white arrowhead in panel B). The plasma in the capillaries shows relatively intense electron density resulting from heavy metal staining (asterisks in panels A and B).

C-G EM showing details of stria in *Ndp*-KO at 1 month.

C Arrow indicates loose fibrils in the space close to capillary

D A cell adjacent to a capillary appears to be shrinking.

E Abnormal marginal cell. The cell has lost intense electron dense staining, and its baso-lateral infoldings, appearing to have become more rounded. The nucleus is more rounded in shape than its usual elongated morphology.

F EM showing marginal cells at 2 months. Cells have lost intense electron dense staining and nucleus is abnormally shaped. Unusual projections from luminal surface suggest possible cell shrinkage as harbinger of possible degeneration.

G-L No abnormalities were observed in endothelial tight junctions (arrows) in strial capillaries of *Ndp*-KO animals at 1 month.

N = 6 WT, N = 6 *Ndp*-KO were analysed at 1 and 2 months.

Scale bars = 0.2 µm (B), 0.5 µm (C) 2 µm (A,D,E,G), 5 µm (F) 1 µm (H, K).

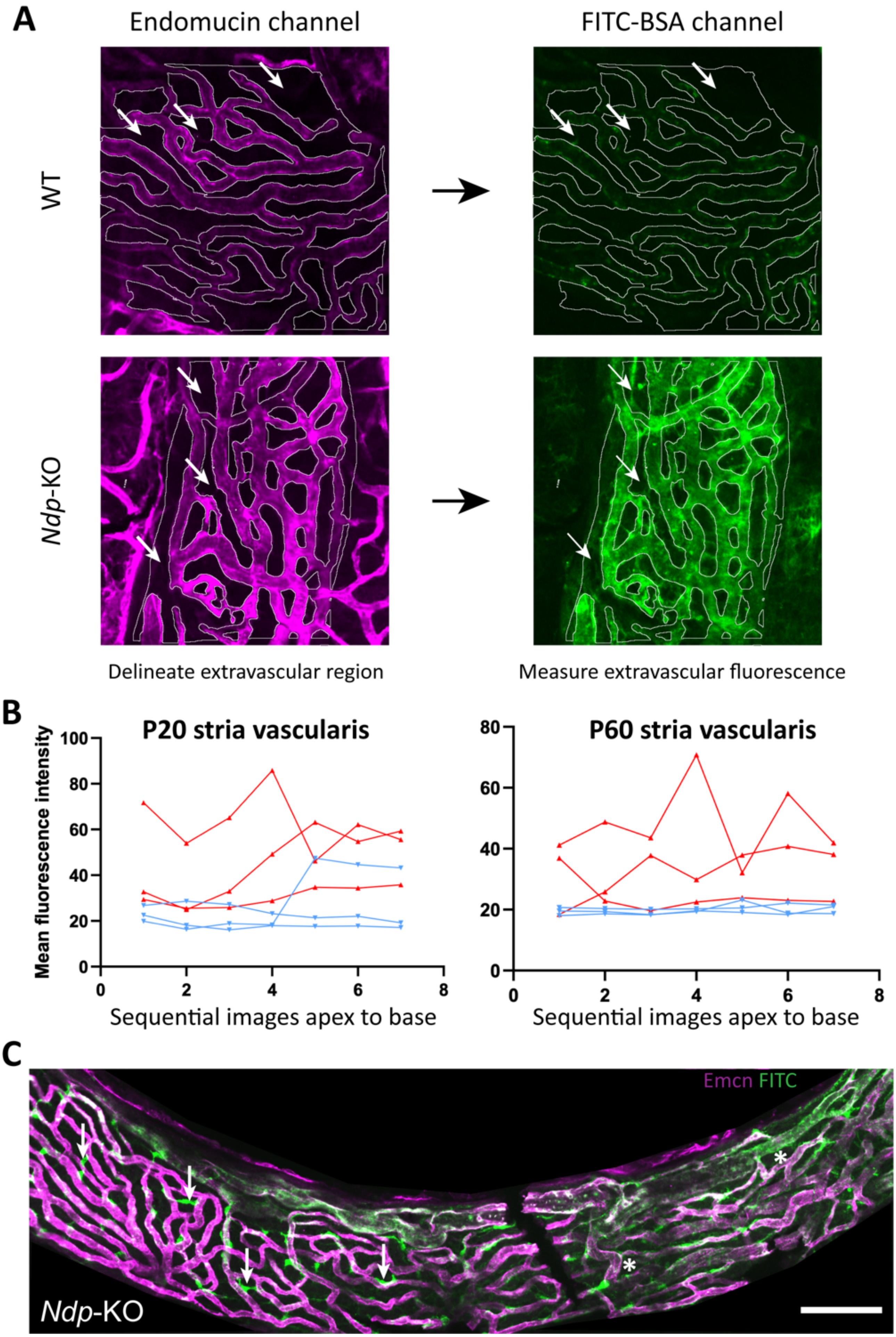
A
Endomucin channel
FITC-BSA channel
WT
Ndp-KO
Delineate extravascular region
Measure extravascular fluorescence
B
P20 stria vascularis
Mean fluorescence intensity
Sequential images apex to base
P60 stria vascularis
Sequential images apex to base
C
Emcn FITC
Ndp-KO

Supplementary Figure 5. Quantification of extravascular FITC-BSA in the stria vascularis as a measure of vascular leakiness.

Whole mount lateral wall preparations were imaged under consistent conditions and multiple fields of view from the apical and middle regions were analysed.

A Shows the analysis method: the extravascular region was delineated using the Endomucin channel; this outline was overlayed on the FITC channel and mean fluorescence intensity was measured in the extravascular region; N = 3 WT and 3 Ndp-KO mice, over 7 fields of view from each sample.

B Graph of mean fluorescence intensity of individual fields of view shows higher intensity in the *Ndp*-KO and variation along the length of the lateral wall and between samples, indicating likely variability in vascular permeability.

C Whole mount preparation of the *Ndp*-KO lateral wall showing the distribution of extravascular FITC-BSA, variably labelling extravascular cells (arrows) or the extravascular space (asterisks).

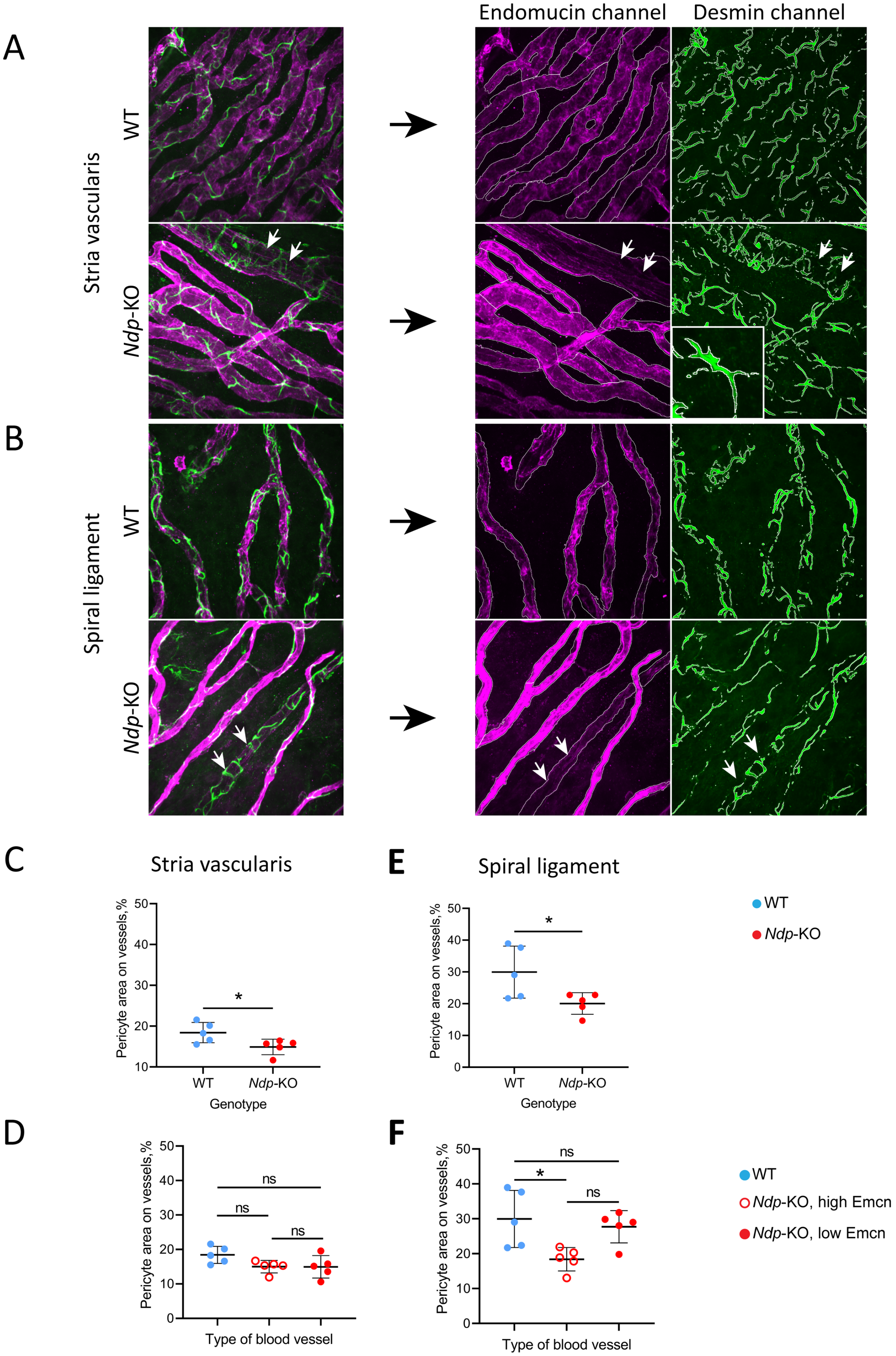
A
B
C
D
E
F
Endomucin channel
Desmin channel
Stria vascularis
Spiral ligament
WT
Ndp-KO
Stria vascularis
Spiral ligament
Pericyte area on vessels,%
Genotype
Type of blood vessel
*
ns
WT
Ndp-KO
Ndp-KO, high Emcn
Ndp-KO, low Emcn

Supplementary Figure 6. Analysis of pericyte coverage in the lateral wall.

Quantification of pericyte coverage of capillaries in the lateral wall affecting the stria vascularis (A) and spiral ligament (B). N=5 WT and N=5 *Ndp*-KO. **A, B** Vessels were delineated in the endomucin channel using a combination of thresholding and manual drawing. Pericytes were delineated using thresholding in the Desmin channel. The percentage area of each vessel segment covered by pericytes was measured and an average of all vessel segments was calculated. Arrows indicate vessel classification as low or high endomucin labelling: with low endomucin vessels showing transversely wrapped pericytes, or high endomucin vessels showing more longitudinally arranged pericytes.

C, D Pericyte coverage in the stria vascularis. Coverage in the *Ndp*-KO was significantly lower than in the WT.

E, F Pericyte coverage in the spiral ligament. Coverage in the *Ndp*-KO was significantly lower than in the WT. When vessels in the Ndp-KO were separated by vessel type, those with high endomucin in the spiral ligament had a significantly lower pericyte coverage.

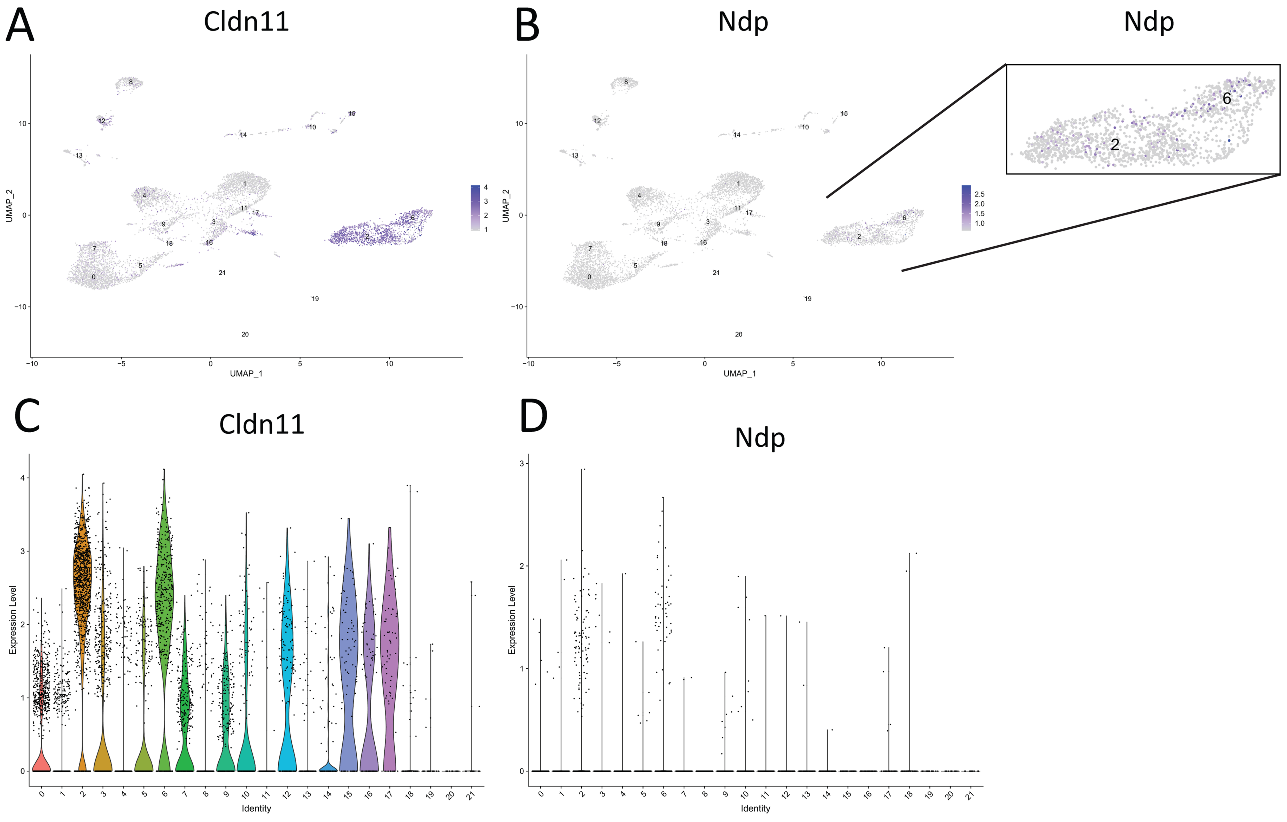

Supplementary Figure 7. Analysis of single cell RNAseq of lateral wall.

A, B UMAP plot showing gene expression at the single cell level in the adult mouse cochlear lateral wall. *Ndp* expression is detectable in a subset of cells within the cluster of basal cells, identified by high *Cldn11* expression. (Gu et al., 2020); GEO data base Accession Number GSM4618125/ GSM4618124).

C, D Violin plots showing high *Cldn11* and *Ndp* expression in clusters 2 and 6.

SUPPLEMENTAL DATA

The Timing of Auditory Sensory Deficits in Norrie Disease has Implications for Therapeutic Intervention

D. Bryant*, V. Pauzuolyte*, N. J. Ingham, A. Patel, W. Pagarkar, L. Anderson, K. E. Smith, D. Moulding, Y.C. Leong, D. Jafree, D. Long, A. Al-Yassin, K. P. Steel, D.J. Jagger, A. Forge, W. Berger, J. C. Sowden◆, M. Bitner-Glindzicz

Supplementary Methods

Animals

Norrie disease mice (*Ndp^{tm1Wbrg}*) generated in the Berger laboratory carried a loss of function *Ndp* mutation, in which exon 2 of the mouse *Ndp* gene is replaced by a neomycin resistance cassette to disrupt the function of the gene [1]. The *Ndp^{tm1Wbrg}* mutation was maintained on a predominantly C57BL/6 genetic background following backcrossing of the 129 founder to the C57BL/6 inbred strain for multiple generations. Genotyping was carried out using forward primer GTATTGCATCCATATTTCTTGG and reverse primer CTCTCCATCCCCTGACAAGGA in a PCR protocol of 94°C for 5 minutes followed by 40 cycles of 94°C for 60 seconds, 62°C for 60 seconds and 72°C for 90 seconds. The reaction finished with a final step of 72°C for 5 minutes. This yielded a 528bp product from the wild type allele and/or a ~1628 bp product from the knockout allele. *Ndp^{tm1Wbrg}* hemizygous males and homozygous females (referred to as *Ndp*-KO) were compared to littermate control mice (*Ndp $^{Y/+,}$ Ndp $^{+/+}$, Ndp $^{+/-}$*) with an identical genetic background (referred to as WT). The *Cdh23^{ahl}* allele (Cdh23c.753A mutation; [2-4]) on the C57BL/6 genetic background does not affect ABR thresholds at the early ages tested in this study [5].

Auditory Brainstem Response (ABR) recordings

Physiology tests were performed under a single session of anaesthesia, using 0.1 ml / 10 g bodyweight intra-peritoneal urethane (20% w/v in water). Brainstem auditory evoked potentials were measured using a previously described method (Ingham et al., 2019). In brief, anesthetised mice were placed on a heated blanket (Harvard Apparatus) inside a sound attenuating chamber (IAC Limited, Winchester, UK) and positioned to face a loudspeaker (FF1 magnetic speaker, Tucker Davis Technologies TDT, Alachua, FL, USA) at a distance of 10 cm. Subdermal needle electrodes [SD51-426-1 NeuroDart, Spes Medica, Italy] were inserted in the skin overlying the right and left bullae (ground and reference electrodes, respectively) and on the midline vertex (active electrode) and were connected to the appropriate input of a TDT RA4LI headstage (with RA4PA preamplifier). Under control of custom software, stimuli were generated, amplified and presented to the FF1 speaker via a TDT RZ6 multifunction processor. The same software and processor also gathered digitised evoked potential recordings from the electrodes via the TDT RA4LI/RA4PA. Acoustic stimuli (broad-band click transients of 10 μs duration, and tone pips of 6, 12, 18, 24, 30, and 42 kHz of 5 ms duration with a 1 ms onset and offset ramp) were presented to the mouse at

levels ranging from 0-95 dB SPL (in 5 dB steps) at a rate of 42.3 stimuli per second. Evoked responses, bandpass filtered at 300 – 3000 Hz, 20 ms in duration, were recorded following the onset of each individual stimulus presentation and were averaged in a digital signal buffer on the TDT RZ6 before being retrieved by the software for online display and saved for offline analyses.

ABR findings in mice aged 1 month were also confirmed using an independent ABR recording apparatus. ABR from anesthetised mice were obtained using subdermal needle electrodes (Rochester Medical), one inserted at the vertex, and one each behind the ipsilateral and contralateral pinnae. Electrode signals were low-pass filtered (7.5 kHz cut-off frequency) and recorded at 24 kHz sampling rate (TDT RA4LI, RA4PA and RX5). For analysis, ABR data were filtered using a bandpass filter (100–3000 Hz). Stimuli were tone pips (10 ms total duration with 1.5 ms rise/fall time; frequencies 5.7, 8, 11.3, 16, 22.6, 32 and 45.3 kHz) or clicks (50 µs duration), with intensities 0–80 dB SPL in 5 dB steps, delivered at a rate of 25/s.

ABR thresholds were determined visually by estimating the lowest sound level at which at least two deflections in the ABR waveform were greater than the background variability in the waveforms. Measurements of wave amplitudes were performed using custom Matlab software. The user selected a time window containing the wave of interest, and the software then detected maxima and minima of the ABR traces within that window. ABR wave I amplitudes were measured from the peak to the following trough.

Distortion Product Otoacoustic Emissions (DPOAE) recordings

After completion of the ABR measurements mice were laid on their right side and their left pinna was removed. A hollow perspex conical speculum was used as a couple between the ear canal and the DPOAE measurement probe; an Etymotic ER10B+ system (Etymotic Research Inc, Elk Grove Village, IL, USA) coupled to TDT MF1 loudspeakers via 5 cm tubes. Stimuli were generated and DPOAE responses recorded using the TDT RZ6 multifunction processor, under the control of TDT BioSigRZ software. Stimulus tones, f1 and f2 were generated on independent output channels. Frequencies for f2 were set to match some of the ABR tone-pip frequencies used (6, 12, 18, 24 and 30kHz). Frequencies for f1 tones were set such that f2 = 1.2 x f1. Sound pressure levels of the f2 stimulus ranged from –10 dB to 65 dB in 5 dB steps and were –10 dB relative to the SPL of the f1 component. ER10B+ microphone signals during stimulus presentation were recorded via the RZ6 processor. Online Fast Fourier Transformation of the microphone signal revealed a power spectrum that contains the f1 and f2 stimulus components, the main 2f1-f2 DPOAE component of interest and the measurement noise-floor. These spectra were exported for analysis.

Endocochlear Potential (EP) recordings

Following completion of DPOAE recording, a tracheal cannula was inserted, and the mouse placed in a custom-built head-holder. An Ag-AgCl pellet was inserted into the musculature of the neck to

serve as a reference / ground electrode for the measurement. Muscle and tissue were surgically retracted using forceps to expose the wall of the auditory bulla. After opening the bulla, a small hole was made in the bone of the lateral wall of the basal turn of the cochlea and the tip of a 150 mM KCl-filled glass micropipette electrode was positioned to be in fluid contact with the spiral ligament tissue within the hole. A custom-built electrometer was zeroed, and the microelectrode was slowly advanced through the spiral ligament under manual control. The electrometer potential was monitored until a stable positive potential, the endocochlear potential (EP) was reached as the electrode entered the scala media [5-7].

Fixation and decalcification of cochleae

Auditory bullae were isolated, and the cochleae exposed and fixed by perfusion of 4% paraformaldehyde (PFA) injected directly via the round and oval windows. Fixation was continued by immersion of the cochlea in PFA for 90-120 mins followed by decalcification in 4% EDTA in PBS (w/v), pH 7.4, for 48 h.

3D imaging of cochlear vasculature

Fixed, decalcified cochleae were dehydrated in sequential stages of 25%, 50%, 75% and 100% methanol for 1 hour each. They were then incubated in 1.5% hydrogen peroxide (Sigma H1009) solution made up in methanol overnight at 4°C on a roller. The following day they were rehydrated in sequential solutions each lasting 1 hour: 75%, 50% and 25% methanol, PBS 0.2% Triton X-100. Cochleae were permeabilised (0.2% Triton X-100, 20% DMSO in PBS) at 4°C for three days. The cochleae were then blocked (0.2% Triton X-100, 10% DMSO, 10% FBS, and 3% BSA in PBS) for 6 hours at room temperature on a roller. They were then incubated with a rat anti-endomucin antibody (Santa Cruz, sc-53941; at a dilution of 1:50 made up in a buffer of PBS, 0.2% Tween 20, 5% DMSO, 10% FBS, 3% BSA) for 4 days at 4°C on a roller. This was followed by six washes in PBS with 0.2% Tween 20, each lasting an hour at room temperature on a roller. The cochleae were then incubated with secondary antibody (goat anti-rat IgG Alexa Fluor 647; Life Technologies, A221247; with 0.2% Tween 20, 5% DMSO, 10% FBS, 3% BSA in PBS) for 3 days at 4°C on a roller. This was followed by six washes in PBS 0.2% with Tween 20, each lasting an hour at room temperature on a roller before a final step in the same solution overnight at 4°C on a roller. They were then dehydrated in sequential stages of 25%, 50%, 75% and 100% methanol for 1 hour each. The cochleae were cleared and mounted in BABB (1 part benzyl alcohol (Sigma, 305197) / 2 parts benzyl benzoate (Sigma, B6630) before imaging on a laser scanning confocal microscope (Zeiss, LSM880).

Morphometric analysis of stria vascularis

The stria vascularis was isolated from the apical region of fixed and decalcified cochleae. Samples were permeabilised and blocked (0.3% BSA, 0.5% Triton X-100 in PBS) for 30 minutes at room

temperature. They were then incubated with GS-IB4 Alexa Fluor 594 at a dilution of 1:50 (ThermoFisher, I21413; 0.3% BSA, 0.1% Triton X-100, 1mM $CaCl_2$ in PBS) for three days at 4°C. This was followed by six washes of 15 minutes each before mounting in Fluoroshield (Sigma, F6182), and samples were imaged using a spinning disk confocal microscope (Yokogawa, CSU22).

Image analysis was carried out using FIJI/ImageJ [8]. Vascular morphology was determined using GS-IB4 fluorescence. Vessels were delineated using the drawing tool and shape descriptors were recorded. Circularity (4π*area/perimeter^2) and solidity (area/convex) were calculated. Vessel diameter was determined at their middle section (i.e. approximately equal distance between two branching points) using the line tool.

Immunostaining of whole mount lateral wall and organ of Corti

The otic capsule was carefully removed from fixed, decalcified cochleae, and the lateral wall was separated from the organ of Corti with microscissors. Samples were blocked for 1 hour at room temperature (1% BSA, 0.5% Triton X-100, 5% FBS in PBS), stained with primary antibodies or phalloidin-A647 at appropriate dilutions (1% BSA, 0.5% Triton X-100, 5% FBS in PBS) followed by 3 washes of 10 minutes each in PBS, and then stained with secondary antibodies at a dilution of 1:250 (1% BSA, 0.5% Triton X-100, 5% FBS in PBS) followed by 3 washes of 10 minutes each in PBS. The following primary antibodies were used: anti-endomucin (1:100 Sc53941), anti-desmin (1:300, 165201-AP), anti-myo7A (1:100, 25-6790, Proteus Biosciences), anti-ZO-1 (1:100 61-7300; ThermoFisher) followed by secondary antibodies at dilution as indicated in Supplementary Table1: goat anti-mouse IgG Alexa Fluor 488 (A11001; Life Technologies), donkey anti-mouse IgG Alexa Fluor 594 (A21203; Life Technologies), goat anti-rat IgG Alexa Fluor 647 (A21247; ThermoFisher), donkey anti-rabbit IgG Alexa Fluor 488 (A21206; Life Technologies), donkey anti-rabbit IgG Alexa Fluor 555 (A31572; ThermoFisher), goat anti-rabbit IgG Alexa Fluor 568 (A11036; ThermoFisher), goat anti-chicken IgY Alexa Fluor 555 (A21437; Life Technologies). The stria was mounted intact, while the organ of Corti was cut into 5-6 pieces, remaining modiolus and lateral wall tissues trimmed and mounted (ProLong™ Gold Antifade Mountant) orientating pieces in an apex-to-base direction. Regions and features of the lateral wall (stria vascularis, spiral ligament vasculature and filopodia, marginal cells and pericytes), at the apex and middle region were imaged using a spinning disk confocal microscope (Yokogawa, CSU22) or fluorescence microscope (Zeiss Observer).

Image analysis for pericyte coverage was carried out using Image-J, based on a modification of a previously published protocol [9]. Z-stack images were acquired from the apical and middle regions of cochlear lateral wall wholemounts labelled for endomucin (endothelial cells) and desmin (pericytes) using a 40x objective and z-projections prepared. Single channel images were thresholded to delineate pericytes and the outlines of vessels were delineated using Image-J

drawing tools. Pericyte coverage was calculated as the percentage of desmin-labelled area to the endomucin-labelled area within the total vessel outlines. Vessel coverage was also analysed after classification of vessels as either those with low endomucin and transversely wrapped pericytes (low Emcn), or those with high endomucin and longitudinally arranged pericytes (high Emcn).

Electron microscopy

The cochlear tissues were fixed by direct perfusion with 2.5% glutaraldehyde in 0.1 M cacodylate buffer, pH 7.35 with 3 mM $CaCl_2$ via the round and oval windows with a hole punctured in the apex to aid immersion. The entire opened bulla was then immersed in that fixative and maintained for 24 h at 4°C. After decalcification with 4% EDTA (w/v) prepared in cacodylate buffer for 72 h, the bulla was trimmed and the entire, intact cochlea was post-fixed in 1% OsO_4 in cacodylate buffer. The samples were partially dehydrated in an ethanol series to 70% ethanol, incubated overnight in uranyl acetate in 70% ethanol before completion of dehydration, and then embedding in plastic. Sections of the entire cochlea were cut parallel to the modiolus and partial serial sections collected onto silicon wafers. The sections were stained with uranyl acetate and lead citrate. The silicon wafers were mounted on scanning electron microscope (SEM) support stubs using conductive silver paint and the samples were then coated with carbon by evaporation to provide a conducting layer ca. 7.5 nm thick. The samples were examined and imaged in a JEOL 6700F SEM using backscatter detection. This procedure enables examination in a single section of an entire cochlea - basal to apical turns and of all tissues - without the fields of view interrupted by the grid bars of the section supports used in transmission electron microscopy. The images collected have been reverse contrasted to provide “photographic positive” contrast for display.

The cross-sectional areas of all strial capillaries in a single section of the entire cochlea of every animal were measured by tracing around the circumference of the lumen using Image-J. The thickness of the stria, from the luminal surface to the interface between the basal aspect of the basal cell and the spiral ligament, at its mid-point between the Reissner’s membrane and the spiral prominence was measured for all cochlear turns in a single section of the entire cochlea for every animal using Image-J.

Vascular permeability assay

Fluorescein isothiocyanate-conjugated bovine serum albumin (FITC-BSA; A9771, Sigma-Aldrich) was dissolved in PBS at 5% concentration (w/v). 50 µl or 100 µl of solution was injected into the tail vein of 1- or 2-month-old mice, respectively. After 5 hours, mice were sacrificed, cochleae isolated and the vasculature counter-stained with an anti-endomucin antibody for analysis, as described above.

Image analysis for extravascular FITC-BSA signal intensity was carried out using a custom Image-J macro. Stitched tile scan images or the stria vascularis were obtained from the apical and middle

regions of cochlear lateral walls from FITC-BSA injected mice as described above. Fields of view of equivalent areas were analysed from WT and *Ndp*-KO mice. The extravascular regions were delineated using a combination of thresholding and manual tracing of vessels. This mask was then superimposed on the FITC channel of the image and the mean grey value of each region was measured and an area weighted mean was calculated.

qRT PCR analysis

Auditory bullae were isolated, the cochleae exposed and separated from the vestibule, immediately snap frozen on dry ice and stored at -80°C .Total RNA was extracted using a modified protocol combining TRIZOL and column based extraction [10]. cDNA was synthesized using the RevertAid First Strand cDNA Synthesis Kit (Thermo Fisher K1621). qPCR quantification was carried out using the PowerSYBR Green PCR Master Mix (4368706). 2-way ANOVA with Sidak's *post hoc* test for pairwise comparisons were performed on dCt values. Fold changes were calculated relative to housekeeping gene Actb and normalized to WT average at each time point. Primers Cldn5 F 5'TTAAGGCACGGGTAGCACTCACG3', Cldn5 R 5'TTAGACATAGTTCTT CTTGTCGTAATCG3', Plvap F 5'GTGGTTGGACTATCTGCCTC3', Plvap R 5'ATAGCGGCGATGAAGGCGA3', Cav1F 5'GCGACCCCAAGCATCTCAA3', Cav1 R 5'ATGCCGTCGAAACTGTGTGT3'

Hair cell analysis

Dissected organ of Corti whole mounts at 1 and 2 months were stained with phalloidin, or at 2 months with anti-myo7a (as described above), then imaged with a spinning disk microscope, stitching together single-plane images. Regions were mapped with Measure_line plugin, dividing the length of the organ of Corti into 8 equal pieces. In each piece, three non-overlapping rectangular lengths of 200 µm (ca. 78 outer hair cells, OHC) were selected for quantitative analysis, giving in total 8 datapoints (1/8-8/8) per total length of the cochlea. The percentage of OHC loss per region was calculated at 1 month from the mean number of dead cells (phalloidin-negative empty sockets of Deiters' cells and loss of OHC nucleus) and at 2 months by subtracting the number of myo7a-labeled surviving cells from the average number of OHC in the sampled area (Mean average 78 OHC). Integrity of the organ of Corti was confirmed by DAPI staining of the whole sample. The predicted frequency of each of eight tonotopic regions from apex to base was calculated using a previously published formula: $F(kHz) = (10^{d \times 0.92} - 0.680) \times 9.8$; d - fractional distance from the apex [11, 12]; region 1/8 (3.1-6.1 kHz); region 2/8 (6.1-10.0 kHz), region 3/8 (10.0-15.0 kHz), region 4/8 (15.0-21.6 kHz), region 5/8 (21.6-30.2 kHz), region 6/8 (30.2-41.3 kHz), region 7/8 (41.3-55.9 kHz), region 8/8 (55.9-74.8 kHz).

Immunostaining of spiral ganglion neurons

Fixed and decalcified cochleae were mounted in 4% low melting point agarose. 200 μm sections were prepared using a vibrating blade vibratome (1000 plus system, Intracel). Mid-modiolar sections through the midline of the cochlea were used for immunostaining. Apical, middle and basal SGN were analysed. Sections were permeabilised and blocked (10% FBS, 0.3% Triton X-100 in PBS) for 1 hour. They were then incubated with the primary antibodies: 1:1000 mouse anti-βIII tubulin (Tubb3) (Promega, G7121); 1:400 rabbit anti-Kir4.1 (Alomone Labs, APC-035); 1:200 rabbit anti-Nf200 (Sigma, N4142); with 10% FBS, 0.1% Triton X-100 in PBS) overnight at 4°C. The next day, sections were washed with PBS before incubation with a combination of secondary antibodies, each at a dilution of 1:400 for 2 hours at room temperature. The following combinations were used: donkey anti-mouse IgG Alexa Fluor 594 (Life Technologies A21203) and donkey anti-rabbit IgG Alexa Fluor 488 (Life Technologies A21206); and donkey anti-rabbit IgG Alexa Fluor 555 (A31572). Secondary antibodies were prepared with 10% FBS, 0.1% Triton X-100 made up in PBS. Sections were mounted in Fluoroshield (Sigma, F6182) and imaged using spinning disk confocal microscope (Yokogawa, CSU22).

Image analysis was carried out using FIJI/ImageJ (Schindelin *et al*., 2012). Quantitative analysis of neurons was carried out on Tubb3 or Nf200 immunoreactivity images as described in the results. The multipoint tool was used to count neuronal cell bodies and the drawing tool was used to trace the perimeter of cell bodies to measure neuronal size.

Statistical analysis

The number of mice (N) used for each experiment is stated in the legends. Error bars always represent standard deviation (SD). Data were analysed using statistical tests as appropriate for each data set. The type of statistical tests, significance levels (*p*-values) *post hoc* analysis are presented in the respective figure legends using either a two-tailed Student's *t*-test or one- or two-way ANOVA with respective *post hoc* tests, or as indicated for auditory function tests using Mann-Whitney Rank Sum Test. P value less than 0.05 was considered significant. Data, graphs and figures were organized, analysed, and assembled using GraphPad Prism 6, SigmaPlot, R (ggplot2), Adobe Illustrator and Inkscape (https://inkscape.org).

Study Approval

Informed consent was obtained from parents or guardians of patients prior to participation. The study was approved by the National Research Ethics Committee London Queens Square (identifier, 17/LO/0841; Understanding the clinical features in patients with Norrie Disease). Hearing test results were examined from 6 patients with a diagnosis of Norrie disease and hearing loss. In two children, information about the newborn hearing screen was available, and they passed this test in both ears. In five out of the six patients, the first available audiogram was recorded at the age of 2-6 years and in one patient at the age of 35 years. The period over which audiograms were available in individual patients, varied from 1-30 years. The British Society of

Audiology classification of hearing loss was used to describe the hearing loss: Mild: hearing levels 21 ≤ 40dBHL; Moderate: 41, ≤ 70 dBHL; Severe: 71, ≤ 95dBHL; Profound: > 95 dBHL

Animal studies were carried out after UCL and King's College London Ethics Review and in accordance with UK Home Office regulations and the UK Animals (Scientific Procedures) Act of 1986 (ASPA) under UK Home Office license.

Analysis of single cell RNASeq datasets

RNAseq data [GEO data base Accession Number GSM4618125/ GSM4618124] was analysed using Seurat V3 [13] in R. Only cells that expressed more than 200 and fewer than 6000 genes, with mitochondrial gene percentages below 60% were considered for analysis. In total, 8293 single cells were analysed. The FindConservedMarkers function in Seurat was used to identify top genes in each cluster identified. UMAP plots showing the expression of *Cldn11* and *Ndp* were plotted using the FeaturePlot function.

1. Berger, W., et al., *An animal model for Norrie disease (ND): gene targeting of the mouse ND gene.* Hum Mol Genet, 1996. **5**(1): p. 51-9.
2. Noben-Trauth, K., Q.Y. Zheng, and K.R. Johnson, *Association of cadherin 23 with polygenic inheritance and genetic modification of sensorineural hearing loss.* Nat Genet, 2003. **35**(1): p. 21-3.
3. Liu, S., et al., *A mutation in the cdh23 gene causes age-related hearing loss in Cdh23(nmf308/nmf308) mice.* Gene, 2012. **499**(2): p. 309-17.
4. Johnson, K.R., et al., *Effects of Cdh23 single nucleotide substitutions on age-related hearing loss in C57BL/6 and 129S1/Sv mice and comparisons with congenic strains.* Sci Rep, 2017. **7**: p. 44450.
5. Ingham, N.J., et al., *Mouse screen reveals multiple new genes underlying mouse and human hearing loss.* PLoS Biol, 2019. **17**(4): p. e3000194.
6. Ingham, N.J., et al., *S1PR2 variants associated with auditory function in humans and endocochlear potential decline in mouse.* Scientific Reports, 2016. **6**(1): p. 28964.
7. Steel, K.P. and C. Barkway, *Another role for melanocytes: their importance for normal stria vascularis development in the mammalian inner ear.* Development, 1989. **107**(3): p. 453-63.
8. Schindelin, J., et al., *Fiji: an open-source platform for biological-image analysis.* Nat Methods, 2012. **9**(7): p. 676-82.
9. Shi, X., *Cochlear pericyte responses to acoustic trauma and the involvement of hypoxia-inducible factor-1alpha and vascular endothelial growth factor.* Am J Pathol, 2009. **174**(5): p. 1692-704.

10. Vikhe Patil, K., B. Canlon, and C.R. Cederroth, *High quality RNA extraction of the mammalian cochlea for qRT-PCR and transcriptome analyses.* Hearing Research, 2015. **325**: p. 42-48.
11. Taberner, A.M. and M.C. Liberman, *Response Properties of Single Auditory Nerve Fibers in the Mouse.* Journal of Neurophysiology, 2005. **93**(1): p. 557-569.
12. Müller, M., et al., *A physiological place–frequency map of the cochlea in the CBA/J mouse.* Hearing Research, 2005. **202**(1): p. 63-73.
13. Stuart, T., et al., *Comprehensive Integration of Single-Cell Data.* Cell, 2019. **177**(7): p. 1888-1902 e21.

Supplementary Table 1. Antibodies

**Antibody**	**Host species**	**Type**	**Catalog number (RRID)**	**Company**	**Dilution**
Anti-Endomucin	rat	monoclonal IgG1	Sc53941 (AB_2100038)	SantaCruz	1:100
Anti-ZO-1	rabbit	polyclonal	61-7300 (AB_138452)	ThermoFisher	1:100
Anti-Desmin	rabbit	polyclonal	16520-1-AP (AB_2292918)	Proteintech	1:300
Anty-Myo7a	rabbit	polyclonal	25-6790 (AB_10015251)	Proteus	1:200
Anti-Myelin Basic Protein (MBP)	chicken	polyclonal	AB9348 (AB_11213157)	Millipore	1:200
Anti-Neurofilament 200 (NF200)	rabbit	polyclonal	N4142 (AB_477272)	Sigma	1:200
Anti-βIII tubulin	mouse	monoclonal	G7121 (AB_430874)	Promega	1:1000
Anti-Kir4.1	rabbit	polyclonal	APC-035 (AB_2040120)	Alomone Labs	1:400
**Conjugated primary antibodies**	**Host**	**Type**	**Catalog number (RRID)**	**Company**	**Dilution**
Anti-Claudin5-Alexa Fluor 488	Mouse	Monoclonal, Clone 4C3C2	352588	Invitrogen	1:300
Target/modification	Type/IgG	Host	Catalog number (RRID)	Company	Dilution
Alexa Fluor 647 phalloidin conjugate	-	-	A22287 (AB_2620155)	Life Technologies	1:200
Alexa Fluor 594 isolectin GS-IB4 conjugate	-	-	I21413 (AB_2313921)	Life technologies	1:100
**Secondary antibody**	**Type/IgG**	**Host**	**Catalog number (RRID)**	**Company**	**Dilution**
Anti-mouse Alexa Fluor 488	IgG(H+L)	goat	A11001 (AB_2534069)	Life Technologies	1:500
Anti-mouse Alexa Fluor 594	IgG(H+L)	donkey	A21203 (AB_141633)	Life Technologies	1:500
Anti-rat Alexa Fluor 647	IgG(H+L)	goat	A21247 (AB_141778)	ThermoFisher	1:250
Anti-rabbit Alexa Fluor 488	IgG(H+L)	donkey	A21206 (AB_2535792)	Life Technologies	1:250
Anti-rabbit Alexa Fluor 555	IgG(H+L)	donkey	A31572 (AB_162543)	ThermoFisher	1:500
Anti-rabbit Alexa Fluor 568	IgG(H+L)	goat	A11036 (AB_10563566)	ThermoFisher	1:250
Anti-Chicken Alexa Fluor 555	IgG(H+L)	goat	A21437 (AB_1500593)	Life Technologies	1:500
